# Implications of Clinical Risk Phenotypes on the Management and Natural History of Atrial Fibrillation: A Report From the GLORIA‐AF

**DOI:** 10.1161/JAHA.123.030565

**Published:** 2023-10-17

**Authors:** Giulio Francesco Romiti, Marco Proietti, Bernadette Corica, Niccolò Bonini, Giuseppe Boriani, Menno V. Huisman, Gregory Y. H. Lip, the GLORIA‐AF Investigators

**Affiliations:** ^1^ Liverpool Centre for Cardiovascular Science University of Liverpool, Liverpool John Moores University, and Liverpool Heart and Chest Hospital Liverpool United Kingdom; ^2^ Department of Translational and Precision Medicine Sapienza–University of Rome Rome Italy; ^3^ Department of Clinical Sciences and Community Health University of Milan Milan Italy; ^4^ Division of Subacute Care IRCCS Istituti Clinici Scientifici Maugeri Milan Italy; ^5^ Cardiology Division, Department of Biomedical, Metabolic and Neural Sciences University of Modena and Reggio Emilia, Policlinico di Modena Modena Italy; ^6^ Department of Thrombosis and Hemostasis Leiden University Medical Center Leiden the Netherlands; ^7^ Danish Center for Clinical Health Services Research, Department of Clinical Medicine Aalborg University Aalborg Denmark

**Keywords:** anticoagulation, atrial fibrillation, clinical complexity, elderly individuals, outcomes, Atrial Fibrillation

## Abstract

**Background:**

Clinical risk factors are common among patients with atrial fibrillation (AF), but there are still limited data on their association with oral anticoagulant (OAC) treatment patterns and major outcomes. We aim to analyze the association between clinical risk phenotypes on AF treatment patterns and the risk of major outcomes.

**Methods and Results:**

The GLORIA‐AF (Global Registry on Long‐Term Oral Antithrombotic Treatment in Patients With Atrial Fibrillation) phase 2 and 3 registries enrolled patients with a recent diagnosis of AF between 2011 and 2016. We defined 4 features of clinical risk among patients with CHA_2_DS_2_‐VASc ≥2: elderly individuals (aged ≥80 years), chronic kidney disease (estimated glomerular filtration rate <45 mL/min), history of stroke, and history of bleeding. We analyzed the odds of receiving OAC and the risk of OAC discontinuation and adverse events at follow‐up according to specific combinations and cumulative burden of these features. Primary outcome was the composite of all‐cause death, thromboembolism, and major bleeding. Among 28 891 (mean±SD age, 70.1±10.5 years; 45.5% women) patients included, 10 797 (37.3%) had at least 1 clinical risk feature. OAC use was lower among patients in the elderly group (odds ratio [OR], 0.85 [95% CI, 0.75–0.96]), those with history of both stroke and bleeding (OR, 0.45 [95% CI, 0.35–0.56]), and those with multiple features (OR, 0.71 [95% CI, 0.62–0.82]). Increasing burden of clinical risk features was associated with OAC discontinuation, with highest magnitude in those with ≥3 features (hazard ratio [HR], 1.68 [95% CI, 1.31–2.15]). Groups with increasingly complex clinical risk phenotypes were associated with the occurrence of the primary composite outcome, with the highest figures observed for groups with a history of both stroke and bleeding (adjusted HR, 2.36 [95% CI, 1.83–3.04]) and multiple features (adjusted HR, 2.86 [95% CI, 2.52–3.25]).

**Conclusions:**

In patients with AF, clinical risk phenotypes are multifaceted and heterogenous, and they are associated with differences in stroke prevention and worse prognosis.

**Nonstandard Abbreviations and Acronyms**
GLORIA‐AF: Global Registry on Long‐Term Oral Antithrombotic Treatment in Patients With Atrial Fibrillation
NOAC: non–vitamin K antagonist oral anticoagulant
OAC: oral anticoagulant
VKA: vitamin K antagonist

**Clinical Perspective**

**What Is New?**

- In patients with atrial fibrillation, complex clinical risk phenotypes are common and are associated with lower use of oral anticoagulant.
- Patients with atrial fibrillation and complex phenotypes showed higher risk of major adverse outcomes, with risk influenced by the specific combination of risk factors.

**What Are the Clinical Implications?**

- The combination of multiple risk factors is common in patients with atrial fibrillation and leads to complex phenotypes, being associated with different management.
- The presence of multiple risk factors was associated with worse prognosis, including higher risk of all‐cause mortality, thromboembolism, and major bleeding.
- Patients with more complex phenotypes may require further improvements in the management to improve their prognosis.

With the progressive aging of the population, incidence and prevalence of atrial fibrillation (AF) are increasing, and are projected to increase steadily over the next decades.^1^, ^2^ Consistently, a significant number of patients with AF currently present with a high burden of comorbidities,^3^ and, in turn, a higher risk of thromboembolism, bleeding, and death. The heterogeneous interplay between different conditions and risk factors commonly entails the definition of “clinical complexity,” wherein the interaction of aging, comorbidities, and risk modifiers poses significant challenges in the management and treatment of these patients, and results in unpredictable risks of adverse events.^4^, ^5^

Older age, chronic kidney disease (CKD), and history of thromboembolic and bleeding events are among the most influential features of increasing clinical complexity, which exert detrimental effects on prognosis.^5^, ^6^ Although these patients usually present with high CHA_2_DS_2_‐VASc score and have, therefore, an indication for oral anticoagulant (OAC),^5^, ^7^ more complex clinical risk phenotypes are a significant driver of bleeding risk in patients treated with OAC, thus potentially leading to undertreatment, suboptimal stroke prevention, and ultimately worse prognosis.^8^, ^9^, ^10^, ^11^, ^12^ Notably, patients with AF with complex phenotypes are often underrepresented in randomized clinical trials, and, as we previously showed, our understanding of the relationship between complexity features, OAC treatment, and prognosis of AF is currently limited.^13^

In this analysis, using data from the GLORIA‐AF (Global Registry on Long‐Term Oral Antithrombotic Treatment in Patients With Atrial Fibrillation) phase 2 and 3, we evaluated the following: (1) the combination of 4 clinical risk factors (as defined by age, chronic kidney disease, history of bleeding, and history of stroke) into clinically meaningful groups of patients; (2) the association between these groups and OAC use and discontinuation, as well as the risk of major adverse outcomes; and (3) the cumulative burden of clinical risk factors and its associations with OAC management and the risk of major outcomes.

## METHODS

This publication is based on research using data from data contributors Boehringer Ingelheim that has been made available through Vivli, Inc. Vivli has not contributed to or approved, and is not in any way responsible for, the contents of this publication.

### Study Population

The GLORIA‐AF is a global, multicenter prospective registry structured in 3 phases, which aimed to evaluate the long‐term safety and effectiveness of dabigatran in real‐world patients with AF. Details on the design and rationale of the GLORIA‐AF study have been previously reported,^14^, ^15^ as well as the primary analyses comparing dabigatran versus vitamin K antagonist (VKA) and other non–vitamin K antagonist oral anticoagulants (NOACs).^16^, ^17^ The protocol of the study was approved by the European Medicines Agency, and the study was conducted following the principles of Good Clinical Practice and the Declaration of Helsinki. Local institutional review boards at each participating site gave ethical approval. In this analysis, we used data from the phase 2 and phase 3 of the GLORIA‐AF program.

### Inclusion Criteria and Procedures

Complete details on inclusion and exclusion criteria are reported elsewhere.^17^ Patients aged ≥18 years, with a recent diagnosis of AF (<3 months, except in Latin America, where a <4.5‐month cutoff was used) and a CHA_2_DS_2_‐VASc score≥1, who provided written informed consent were considered eligible for inclusion, and were enrolled between 2011 and 2014 for phase 2 and 2014 and 2016 for phase 3.

At baseline, data on age, sex, type of AF (paroxysmal, persistent, or permanent), comorbidities, and CHA_2_DS_2_‐VASc scores were collected. All patients recruited were treated according to local clinical practice, and treatment choices were at the discretion of the treating physician.

For this analysis, we included only patients with complete data on the conditions evaluated (chronic kidney disease, elderly individuals, history of stroke, and history of bleeding; see below).

### Definition of Features, Groups, and Burden

For this analysis, we analyzed clinical risk profile phenotypes according to *features* (ie, single characteristics underpinning clinical complexity among patients with AF), *groups* (ie, different combinations of features), and *burden* (ie, the cumulative number of features).

To focus on patients with already high thromboembolic risk, we analyzed clinical risk phenotypes among patients with CHA_2_DS_2_‐VASc ≥2, whereas those with CHA_2_DS_2_‐VASc <2 were considered as a separate group. Among patients with CHA_2_DS_2_‐VASc ≥2, we initially identified 4 features of clinical risk, with impact on AF outcomes:

- *CKD*: patients with an estimated glomerular filtration rate (eGFR) <45 mL/min (category 3B or higher according to Kidney Disease: Improving Global Outcomes CKD nomenclature^18^). For this analysis, eGFR was calculated according to the baseline serum creatinine levels, using the CKD Epidemiology Collaboration 2021 formula.^19^
- *Elderly individuals*: aged ≥ 80 years.
- *History of stroke*: patients with previous history of ischemic stroke or transient ischemic attack, as reported in the case report form by the investigator.
- *History of bleeding*: patients with previous history of bleeding, as reported in the case report form by the investigator.

As these features often coexist and occur concomitantly, we further defined 8 *groups* of clinical risk phenotypes, according to the following combinations of clinical features, so that each patient can only be included in 1 group:

- *CHA*_*2*_ *DS*_*2*_ *‐VASc* <*2 group*: those with CHA_2_DS_2_‐VASc <2, regardless of the presence of features;
- *CKD group*: those with CHA_2_DS_2_‐VASc ≥2 and only “CKD” feature;
- *Elderly group*: those with CHA_2_DS_2_‐VASc ≥2 and only “elderly” feature;
- *Stroke group*: those with CHA_2_DS_2_‐VASc ≥2 and only “history of stroke” feature;
- *Bleeding group*: those with CHA_2_DS_2_‐VASc ≥2 and only “history of bleeding” feature;
- *Stroke and bleeding group*: those with CHA_2_DS_2_‐VASc ≥2 and both “history of stroke” and "history of bleeding” features;
- *Multiple features group*: those with CHA_2_DS_2_‐VASc ≥2 and all the other combinations of ≥2 features (excluding those with both history of stroke and history of bleeding);
- *CHA*_*2*_ *DS*_*2*_ *‐VASc ≥2 group*: those with CHA_2_DS_2_‐VASc ≥2, without any other feature.

The CHA_2_DS_2_‐VASc ≥2 group was taken as the reference group for all the analyses.

We finally defined the *burden* of clinical risk factors, in relation to the number of clinical features found in each patient (0, 1, 2, and ≥3 clinical features). We analyzed the burden following 2 approaches: (1) according to the total number of clinical features and (2) conditionally to each feature (ie, as the number of additional features other than the one considered).

### Follow‐Up, Persistence, and Major Adverse Outcomes

Descriptions of follow‐up and outcomes for phase 2 and phase 3 were reported elsewhere.^17^, ^20^ Briefly, a 2 year follow‐up was performed for patients enrolled in phase 2 who initiated dabigatran, whereas all patients enrolled in phase 3 (irrespective of the antithrombotic treatment received) were followed up for 3 years. During follow‐up, data on OAC discontinuation and major adverse outcomes were collected, until study withdrawal, death, or end of the study. We defined OAC nonpersistence as either discontinuation or study termination. We defined discontinuation as either switching to another antithrombotic regimen (including switching to a different OAC) or a ≥30‐day interruption of the treatment received at baseline (to exclude temporary interruptions attributable to invasive procedures or surgery). Dose adjustments/reductions were not counted as discontinuation. In this analysis, we evaluated nonpersistence and discontinuation at 24 months only for patients who received OAC (either VKA or NOAC) at baseline.

To evaluate the impact of clinical risk phenotypes on the prognosis of patients with AF, we defined our *primary outcome* as the composite of all‐cause death, thromboembolism (including stroke, transient ischemic attack, and other thromboembolism), and major bleeding (defined as a bleeding associated with a reduction in hemoglobin of ≥20 g/L or leading to ≥2 units of blood or packed cell transfusion, a symptomatic bleeding in a critical organ, or life‐threatening/fatal bleeding). We also investigated the following exploratory secondary outcomes: the composite of all‐cause death and major adverse cardiovascular events (including cardiovascular death, stroke, and myocardial infarction); all‐cause death; cardiovascular death; major adverse cardiovascular events; thromboembolism; and major bleeding.

### Statistical Analysis

Baseline characteristics were reported as mean and SD for continuous variables, and comparisons were performed using *t* test or ANOVA. Categorical variables were reported using frequencies and percentages and were compared using Pearson χ^2^ test.

The association between groups and burden of clinical risk phenotypes and use of OAC was evaluated through multiple logistic regression analysis; results were reported as odds ratio (OR) and 95% CI. Multiple adjusted Cox regression analyses were performed to evaluate the impact of (1) groups of clinical risk phenotypes and (2) burden of features on the risk of OAC discontinuation and major outcomes. Results were reported as hazard ratio (HR) and 95% CI.

Regression models for OAC use and discontinuation were adjusted for age class (<65, 65–74, and ≥75 years), sex, type of AF, and CHA_2_DS_2_‐VASc score. For the risk of outcomes, Cox regression models were adjusted for age class, sex, type of AF, use of OAC, and relevant comorbidities (arterial hypertension, diabetes, coronary artery disease, heart failure, and peripheral artery disease). Kaplan‐Meier curves were also reported for the primary composite outcome, and survival distributions were compared using log‐rank test.

A 2‐sided *P*<0.05 was considered statistically significant. All the analyses were performed using R 4.3.1 (R Core Team 2022, Vienna, Austria).

## RESULTS

A total of 28 891 patients (mean±SD age, 70.1±10.5 years; 45.5% women) with complete data to define clinical risk phenotypes were included in this analysis. Among the clinical features, elderly individual was the most prevalent (5994 [20.7%]).

**Figure 1:**
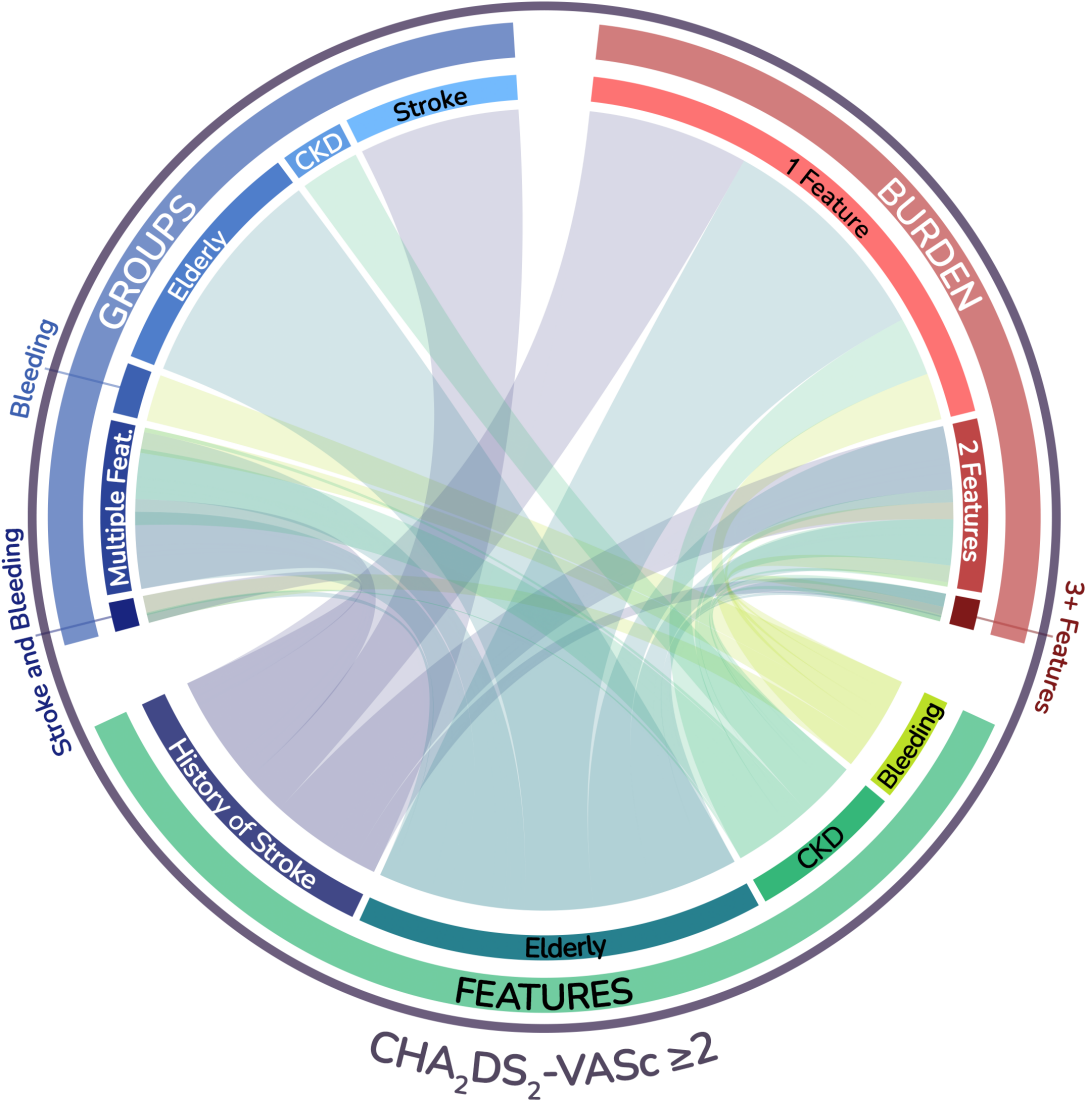
Relationship between features, groups, and burden of clinical risk phenotypes. CKD indicates chronic kidney disease.

The relationship between features, groups, and burden is shown in Figure 1 and Figures S1 and S2. The full representation of the combinations of clinical features, and the resulting groups and burden, is reported in Figure S3.

### Clinical Risk Phenotypes

**Figure 2:**
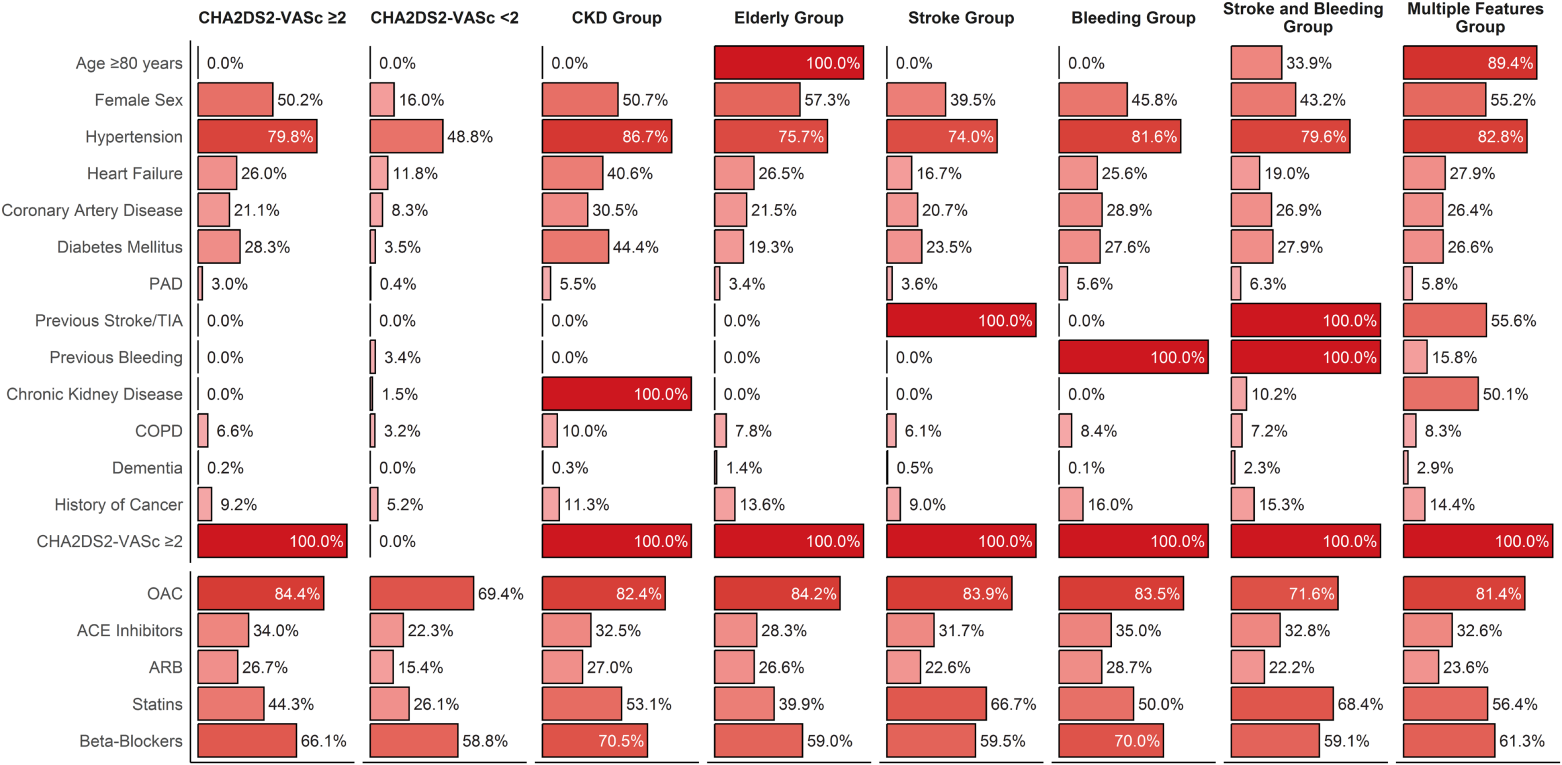
Prevalence of clinical characteristics and relevant treatments according to groups of clinical risk phenotypes. ACE indicates angiotensin‐converting enzyme; ARB, angiotensin‐II receptor blocker; CKD, chronic kidney disease; COPD, chronic obstructive pulmonary disease; OAC, oral anticoagulant; PAD, peripheral artery disease; and TIA, transient ischemic attack.

The most relevant clinical characteristics found in each group analyzed are reported in Figure 2; full baseline characteristics according to groups of clinical risk phenotypes are reported in Table S1. Overall, 14 021 patients (48.5%) were included in the CHA_2_DS_2_‐VASc ≥2 group (ie, without any of the 4 features evaluated), whereas 4073 (14.1%) had a CHA_2_DS_2_‐VASc score of <2 (irrespective of the clinical risk features). Overall, patients with complex clinical risk phenotypes showed higher thromboembolic risk. The elderly group (3563 [12.3%]) was the largest, and with the highest female representation (57.3%); conversely, the stroke group (2558 [8.9%]) was composed mostly of men. The CKD group (942 [3.3%]) showed relevant prevalences of several comorbidities, including heart failure, coronary artery disease, and diabetes, and similar results were observed for the bleeding group (n=746 [2.6%]). Finally, 433 patients (1.5%) were included in the stroke and bleeding group, having both history of thromboembolic and hemorrhagic events, whereas 2555 (8.8%) patients were included in the multiple features group, which was mainly composed of elderly patients with several comorbidities. The highest burden of risk factors was observed among the multiple features and stroke and bleeding groups.

### Use of OAC and Type of OAC Received

Overall, 23 587 (81.6%) patients received OAC at baseline. Use of antithrombotics according to clinical risk phenotype groups is reported in Figure S4. Patients in the stroke and bleeding group were the least treated with OAC (71.6%), with 12.5% who did not receive any antithrombotic treatment at baseline. NOAC uptake was highest among patients in the stroke group (59.3%) and lowest in the CKD group (42.9%).

**Figure 3:**
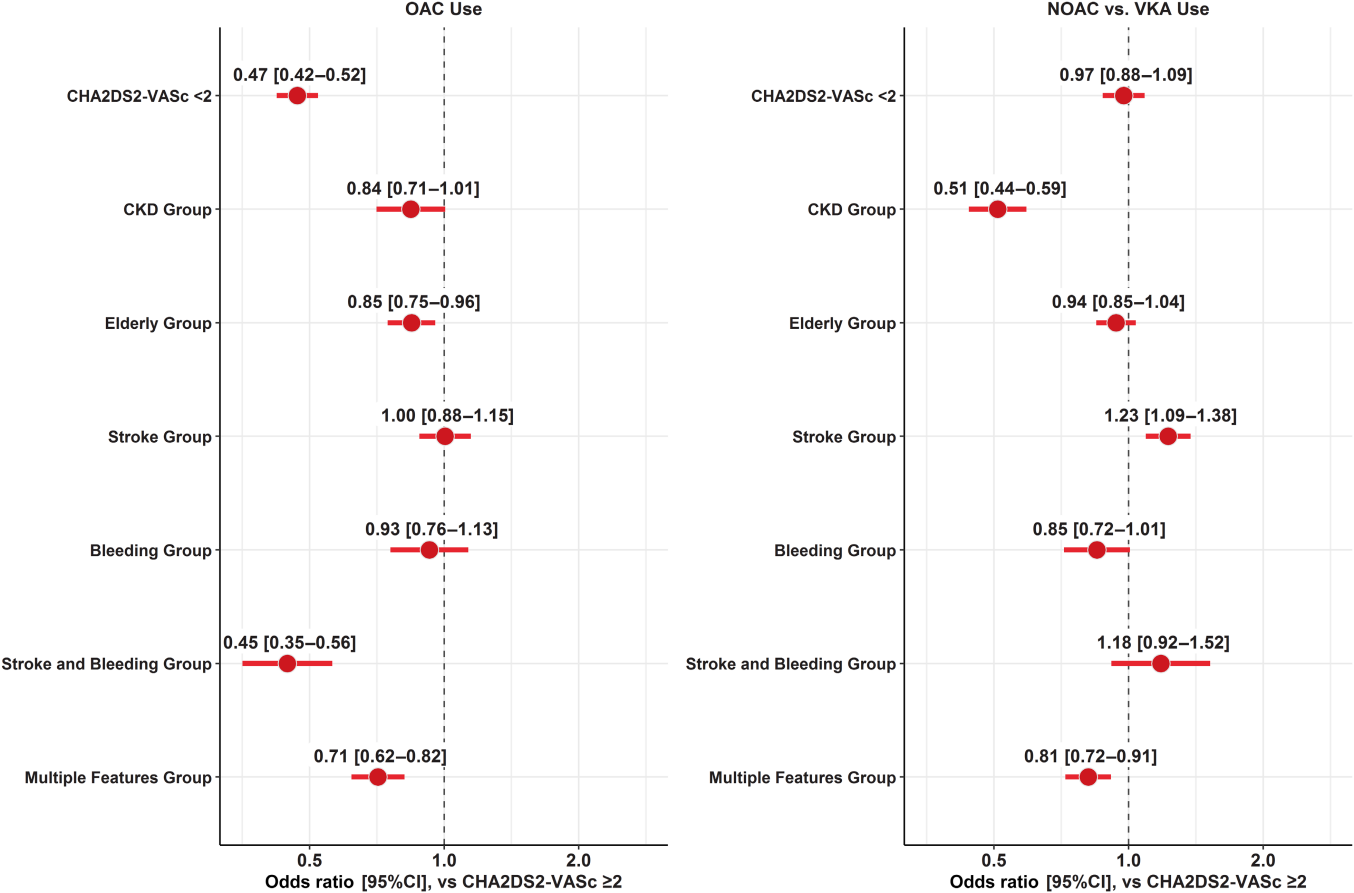
Regression on use of oral anticoagulant (OAC) (left panel) and use of non–vitamin K antagonist oral anticoagulant (NOAC) vs vitamin K antagonist (VKA) (right panel) according to groups of clinical risk phenotypes. CKD indicates chronic kidney disease.

Regression on OAC use is reported in Figure 3. Compared with patients in the CHA_2_DS_2_‐VASc ≥2 group (without risk features), patients in the stroke and bleeding group and multiple features group showed the lowest odds of receiving OAC (OR [95% CI], 0.45 [0.35–0.56] and 0.71 [0.62–0.82], respectively); patients in the elderly group were also less likely to receive OAC (OR [95% CI], 0.85 [0.75–0.96]). Moreover, patients in CKD and multiple features groups were less likely to receive a NOAC compared with VKA (OR [95% CI], 0.51 [0.44–0.59] and 0.81 [0.72–0.91], respectively); conversely, patients in the stroke group showed a higher odds of receiving NOAC (OR [95% CI], 1.23 [1.09–1.38]).

### OAC Discontinuation

Among patients who received OAC at baseline, 17 678 (74.9%) had complete follow‐up data on OAC persistence and discontinuation at 24 months. Rates of OAC persistence and discontinuation at 6 months and 1 and 2 years of follow‐up according to clinical risk phenotype groups are reported in Figures S5 and S6. OAC discontinuation at 2 years was highest in the bleeding and multiple features groups (28.6% and 28.8%, respectively), and lowest in the stroke group (23.3%). Among NOAC users, CKD, bleeding, and multiple features groups showed the highest 2‐year discontinuation rates (27.0%, 26.4%, and 28.5%, respectively); conversely, among VKA users, discontinuation rates were highest in the stroke and bleeding group (38.6% at 2 years).

**Figure 4:**
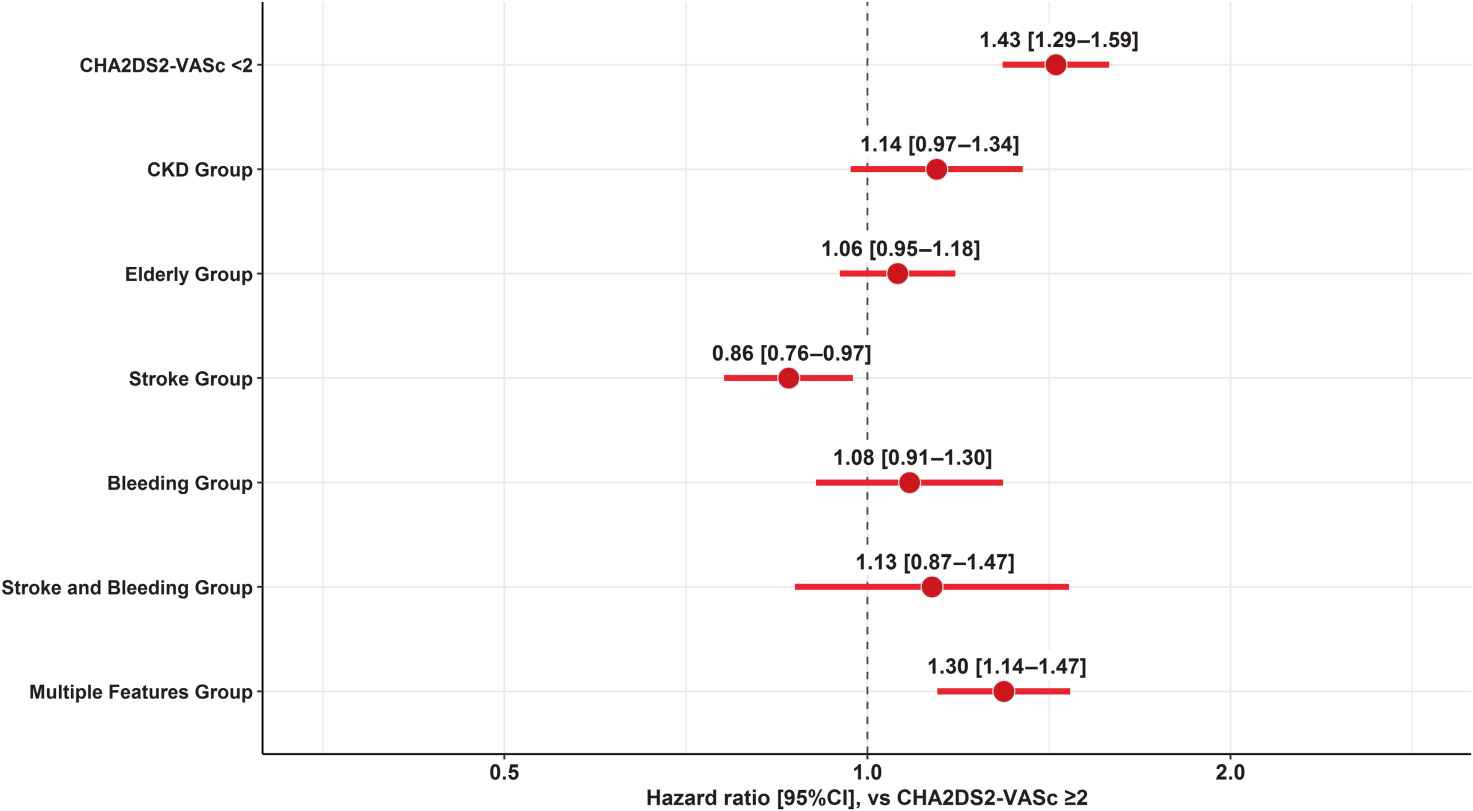
Regression on oral anticoagulant discontinuation according to groups of clinical risk phenotypes. CKD indicates chronic kidney disease.

Cox regression model for OAC discontinuation is reported in Figure 4. Compared with patients in the CHA_2_DS_2_‐VASc ≥2 group without any feature, patients in the stroke group showed lower hazard of OAC discontinuation (HR [95% CI], 0.86 [0.76–0.97]), whereas the multiple features group was associated with higher OAC discontinuation (HR [95% CI], 1.30 [1.14–1.47]), with similar, nonstatistically significant findings observed for all the remaining groups.

We finally analyzed discontinuation according to the type of OAC received at baseline. Broadly consistent results were observed among NOAC users, whereas no statistically significant difference was observed among VKA recipients (Figure S7).

### Impact of Complex Clinical Risk Phenotypes on Prognosis

Overall, 20 521 patients (71.0%) with complete follow‐up data on the primary composite outcome were included in the longitudinal analysis on prognosis. Overall, patients excluded were mainly recruited in phase 2 of the registry (99.1%), with some differences in proportion of women and prevalence of main comorbidities (Table S2).

During a mean (SD) follow‐up of 2.6 (0.8) years, 2761 (13.5%) events of the primary composite outcome were observed, with the multiple features group showing the lowest event‐free survival probability (Figure S8).

Results of the multiple Cox regression models are reported in the Table. Compared with patients in the CHA_2_DS_2_‐VASc ≥2 group (without any feature), rates of the primary composite outcome were higher among all the more complex clinical risk phenotype groups, with the highest magnitude of increase observed in the stroke and bleeding (HR [95% CI], 2.36 [1.83–3.04]) and the multiple features groups (HR [95% CI], 2.86 [2.52–3.25]).

**Table .** Risk of Adverse Outcomes According to Groups of Clinical Risk Phenotypes

Outcome	Groups of clinical risk phenotypes							
	CHA_2_DS_2_‐VASc ≥2 (n=10 168)*	CHA_2_DS_2_‐VASc <2 (n=2846)	CKD group (n=633)	Elderly group (n=2463)	Stroke group (n=1905)	Bleeding group (n=512)	Stroke and bleeding group (n=304)	Multiple features group (n=1690)
Primary outcome								
Composite of all‐cause death, thromboembolism, and major bleeding	3.6 (3.4–3.8), Reference	2.0 (1.7–2.3), 0.89 (0.74–1.09)	9.7 (8.2–11.3), 2.19 (1.83–2.62)†	8.5 (7.8–9.3), 1.83 (1.61–2.08)†	5.8 (5.1–6.5), 1.74 (1.52–1.99)†	4.7 (3.6–6.0), 1.31 (1.01–1.69)†	9.3 (7.2–11.7), 2.36 (1.83–3.04)†	13.4 (12.3–14.6), 2.86 (2.52–3.25)†
Secondary outcomes								
Composite of all‐cause death and MACEs	3.0 (2.8–3.2), Reference	1.6 (1.4–1.9), 0.90 (0.73–1.12)	9.1 (7.6–10.7), 2.34 (1.94–2.81)†	7.6 (6.9–8.3), 1.88 (1.64–2.16)†	4.6 (4.1–5.3), 1.68 (1.45–1.95)†	4.0 (3.0–5.3), 1.29 (0.97–1.72)	7.3 (5.5–9.5), 2.10 (1.58–2.79)†	11.8 (10.7–12.9), 2.87 (2.51–3.29)†
All‐cause death	2.1 (2.0–2.3), Reference	1.1 (0.8–1.3), 0.87 (0.67–1.13)	7.3 (6.0–8.7), 2.61 (2.12–3.21)†	6.2 (5.6–6.8), 2.03 (1.73–2.37)†	2.9 (2.4–3.4), 1.50 (1.24–1.80)†	2.7 (1.9–3.7), 1.19 (0.84–1.68)	5.4 (3.9–7.3), 2.34 (1.70–3.22)†	10.1 (9.1–11.1), 3.30 (2.84–3.84)†
Cardiovascular death	0.8 (0.7–0.9), Reference	0.3 (0.2–0.5), 0.77 (0.48–1.24)	3.2 (2.4–4.2), 3.09 (2.25–4.24)†	2.0 (1.7–2.4), 2.15 (1.64–2.83)†	0.9 (0.6–1.2), 1.29 (0.93–1.79)	1.0 (0.6–1.7), 1.26 (0.72–2.20)	1.9 (1.1–3.2), 2.37 (1.37–4.09)†	4.0 (3.4–4.7), 4.07 (3.16–5.24)†
MACEs	1.7 (1.6–1.9), Reference	0.9 (0.7–1.2), 0.90 (0.67–1.20)	5.2 (4.1–6.5), 2.34 (1.83–3.00)†	3.7 (3.2–4.2), 1.79 (1.48–2.18)†	2.7 (2.3–3.2), 1.73 (1.42–2.10)†	2.3 (1.6–3.3), 1.32 (0.91–1.92)	4.2 (2.9–6.0), 2.09 (1.42–3.07)†	6.1 (5.4–6.9), 2.87 (2.38–3.46)†
Thromboembolism	1.0 (0.9–1.1), Reference	0.6 (0.5–0.8), 0.99 (0.69–1.40)	1.3 (0.8–2.0), 1.11 (0.70–1.78)	1.9 (1.6–2.3), 1.67 (1.29–2.17)†	2.6 (2.1–3.1), 2.78 (2.24–3.46)†	0.8 (0.4–1.5), 0.86 (0.47–1.58)	3.8 (2.6–5.5), 3.39 (2.25–5.10)†	3.2 (2.7–3.8), 2.73 (2.13–3.51)†
Major bleeding	1.1 (0.9–1.2), Reference	0.6 (0.4–0.8), 1.03 (0.71–1.49)	2.6 (1.9–3.6), 2.07 (1.47–2.92)†	1.9 (1.6–2.3), 1.56 (1.20–2.03)†	1.2 (0.9–1.5), 1.18 (0.88–1.58)	1.7 (1.1–2.6), 1.72 (1.12–2.63)†	2.1 (1.2–3.4), 1.99 (1.18–3.37)†	2.7 (2.2–3.3), 2.19 (1.69–2.85)†

Data are given as incidence rate per 100 patient‐years (95% CI), hazard ratio (95% CI). Model adjusted for age classes, sex, type of atrial fibrillation, use of oral anticoagulant, arterial hypertension, diabetes, coronary artery disease, heart failure, and peripheral artery disease. CKD indicates chronic kidney disease; and MACE, major adverse cardiovascular event.

* Patients with CHA_2_DS_2_‐VASc ≥2 and without any other complexity criteria.

^†^ Statistically significant at *P*<0.05.

Among the exploratory secondary outcomes, all the complex clinical risk phenotype groups, except for the bleeding one, were associated with increased hazard of the composite of all‐cause death and major adverse cardiovascular events, as well as with all‐cause death and major adverse cardiovascular events as individual outcomes; similar results were observed for cardiovascular death. Hazard of thromboembolism was highest in patients in the stroke and bleeding group; conversely, all groups were associated with major bleeding, except for the stroke group; the highest magnitude was observed in the multiple features group (Table).

### Burden of Features

Baseline characteristics according to the cumulative burden of the clinical risk factors are reported in Table S3, whereas the cumulative burden in respect to each feature is shown in Figure S9. Among patients with CHA_2_DS_2_‐VASc ≥2, 7809 (27.0%) patients had 1 feature, 2542 (8.8%) had 2 features, and 446 (1.5%) had ≥3 features.

Use of OAC decreased as the burden of features increased, being lowest in patients with ≥3 features (76.5%; Figure S10). Similar results were observed for NOACs, and when analyzing the effect of burden across each feature. Consistently, an increasing burden of features was associated with lower odds of receiving OAC (OR [95% CI], 0.68 [0.60–0.76] for 2 versus 1 feature and 0.48 [0.38–0.60] for ≥3 versus 1 feature; Figure S11); similar results were observed when analyzing the impact of burden across each feature. Increasing burden of features was also associated with lower use of NOAC, and particularly among those with history of stroke.

Rates of OAC discontinuation increased with the burden of features (Figures S12 and S13). Cox regression analyses (Figure S14) showed that a higher cumulative burden of features was associated with higher OAC discontinuation (HR [95% CI], 1.31 [1.16–1.47] for 2 versus 1 feature and 1.68 [1.31–2.15] for ≥3 versus 1 feature); similar results were observed when stratifying the analysis for each feature.

We finally analyzed the association between burden of features and major outcomes. The event‐free survival probability for the primary outcome was lower as the burden of features increased (Figure S15). Consistently, Cox regression models showed that increasing burden of features was associated with a progressively higher hazard of the primary composite outcome (HR [95% CI], 1.44 [1.29–1.61] for 2 versus 1 feature and 2.25 [1.84–2.74] for ≥3 versus 1 feature; Figure S16); moreover, we observed a consistent effect of increasing burden across features, with slightly higher effect among those with bleeding feature (HR [95% CI] for ≥2 additional features: 1.62 [1.17–2.23] and 2.58 [1.79–3.71], respectively).

## DISCUSSION

In this analysis from a large, contemporary global cohort of patients with AF, our principal findings are as follows: (1) clinical risk phenotypes are multifaceted in patients with AF, and complexity features are often found together; (2) clinical risk factors influence both use of OAC and OAC discontinuation, thus underpinning undertreatment and potential suboptimal stroke prevention in patients with AF; (3) prognosis of AF is heterogeneously influenced by clinical complexity, with more complex groups, and greater burden of features, being associated with increased risk of all events, including thromboembolism, bleeding, and death; and (4) an increasing burden of risk factors is a powerful driver of worse prognosis in patients with AF, thus underlying the urgent need for effective strategies to manage these patients and improve their prognosis.

As aging and multimorbidity are increasing among patients with AF, the management of so‐called clinically complex patients has become one of the most pressing unmet needs.^5^ Although several drivers of complexity are already known, the overall impact of the interactions of different features of clinical risk is still poorly understood.

In our article, we identified 4 key features (namely, older age, CKD, history of stroke, and history of bleeding), and we looked at their combinations into clinically relevant clinical risk phenotype *groups*, and at their cumulative *burden*. This approach allowed us to evaluate the multifaceted relationships between clinical complexity, OAC treatment, and prognosis of patients with AF. Indeed, our analysis on OAC use and discontinuation showed how the effect of complex clinical risk phenotypes is heterogeneous in patients with AF: although use of OAC was broadly influenced by most features, we showed how the combination of stroke and bleeding features was associated with the lowest odds of receiving OAC, despite the high thromboembolic risk of these patients. Consistently, use of OAC was largely impacted by the increased burden of features, with an effect that disproportionately affected patients with history of bleeding. The combination of different features also influenced the choice of OAC.

These results show how complex clinical risk phenotypes are associated with OAC undertreatment and, consequently, unsatisfactory stroke prevention among patients at high thromboembolic risk. Although some factors (such as age, history of bleeding, and CKD) have already been associated with lower use of OAC,^21^, ^22^, ^23^, ^24^ we expanded these observations through showing that specific patterns of clinical complexity phenotypes, and the overall burden of features, are even more associated with OAC undertreatment, compared with single features. Although definitive causal associations cannot be proven, these findings suggest that the perceived clinical complexity of patients with AF may represent a significant barrier to OAC use among treating physicians,^25^ with previous evidence that already showed how the attitude toward use of OAC is prominently influenced by the perceived risk of bleeding for particular phenotypes, even when thromboembolic risk is high.^12^, ^26^ Further studies are needed to confirm these hypotheses.

We also found that complex clinical risk phenotypes were associated with risk of OAC discontinuation, and particularly when ≥2 clinical risk features are present; moreover, OAC discontinuation was also associated with the cumulative burden of complexity features. Previous studies showed how the accumulation of deficits and comorbidities represents a key driver of OAC nonpersistence and discontinuation,^27^, ^28^ contributing to undermine the quality of care and, ultimately, the prognosis of patients with AF. Our study further expands these observations, and suggests that discontinuation may be influenced by the clinical risk phenotypes, with higher burden of features potentially associated with a significantly higher risk of unsatisfactory OAC persistence among patients with AF.

Beyond the impact on thromboembolic risk management, our analysis also shows that complex phenotypes are associated with adverse outcomes in patients with AF. Indeed, although all groups showed a significant association with the risk of the primary outcome of all‐cause death, thromboembolism, and major bleeding, we found that the magnitude of association was heterogeneous across groups. Moreover, we also found some differences among the exploratory secondary outcomes, and particularly for the risk of thromboembolism and major bleeding events.

The analysis on the burden of features showed a potential dose‐response relationship effect between the number of features and the risk of the primary outcome, consistently with previous knowledge on the dynamic of risk when >1 risk factor is present, and with the composition of the CHA_2_DS_2_‐VASc score.^29^ Our analysis expands these findings, suggesting that although prognosis of patients with AF is influenced by the cumulative number (ie, the burden) of comorbidities, the specific combinations of features may impose different risks, and should be therefore taken into account to understand and manage the complexity of these individuals.

Taken together, our findings have several clinical implications. First, as clinical risk features often coexist in patients with AF, a better understanding of their synergistic and multiplicative impact on perceived complexity, quality of care, and prognosis is pivotal to mitigate their effect. Second, as the accumulation of features entails worse prognosis, we need specific tools and more comprehensive models to define and capture the overall clinical complexity of these patients. The evaluation of frailty, in this scenario, represents a promising approach^30^; unsurprisingly, the detrimental effects of frailty among patients with AF have already been shown.^31^, ^32^, ^33^ Third, given the association between complex clinical risk phenotypes and lower OAC uptake, higher OAC discontinuation, and high risk of adverse events, approaches to improve management of these high‐risk patients with AF should be developed and implemented in clinical practice.

Indeed, although some specific approaches have been tested in high‐risk subgroups of patients, such as the use of low‐dose edoxaban in elderly patients with AF,^34^ the complexity of these subjects may require a much more holistic and integrated approach. Among the potential interventions to address such complexity, the Atrial Fibrillation Better Care pathway has been proposed to streamline a more comprehensive approach to the care of patients with AF.^35^ The Atrial Fibrillation Better Care pathway is based on 3 pillars: beyond the *A*, avoid stroke through OAC (which still represents the cornerstone of AF management), particular emphasis is placed on the other 2 pillars: *B*, better symptom control; and *C*, cardiovascular and comorbidity optimization. The implementation of the Atrial Fibrillation Better Care pathway is currently recommended by international guidelines,^7^, ^36^ and has already proved effective in reducing the risk of outcomes,^37^, ^38^, ^39^, ^40^ also in clinically complex patients, such as elderly patients and those with multimorbidity.^41^, ^42^, ^43^ In view of this previous evidence, the implementation of such comprehensive and holistic approach to AF care may seem a promising approach to address the challenges imposed by clinical complexity in AF; nonetheless, further studies are required to validate these hypotheses.

### Strengths and Limitations

Our study has several strengths: first, it is based on a large, global, and contemporary real‐world cohort of newly diagnosed patients with AF. Also, the data on OAC management and clinical follow‐up allowed us to evaluate the relationship between different clinical features, treatment patterns, and prognosis of patients with AF, thus providing important observations that are useful to shape our knowledge on the potential areas of interventions.

Nonetheless, we acknowledge some limitations. First, the definition of the clinical risk phenotypes is heterogeneous, and the approach used in this analysis is not exhaustive. We relied on prespecified groups of patients, and applied cutoffs to age and eGFR to define elderly and CKD features. This approach may not totally capture the overall complexity of the interaction between the different features considered, yet it represents a simplistic approach to the analysis of their combination. Nonetheless, we focused on 4 of the most influencing features, and analyzed their combination as both clinically relevant groups and as cumulative burden, and using clinically meaningful cutoffs for age and eGFR. This allowed us to provide a pragmatic, yet insightful, outlook on the combination of these risk factors, although our results should be interpreted with caution, and confirmed by further studies, especially when considering the potential nonlinear effects of age and renal function. Therefore, the interpretation of our results, and particularly those related to the contribution of age and eGFR on the overall complexity of patients with AF, has some limitations and is not conclusive. Second, other factors that may influence OAC treatment and outcomes, including social determinants of health, were not available and analyzed in this study. We have also excluded some patients from the analysis, because of the lack of the relevant data needed to be classified in the complexity phenotypes. Moreover, although we have included multiple covariates in our regression analyses, residual confounding should be considered, and we cannot exclude the contribution of other unaccounted confounders in determining the results observed; therefore, the results should be interpreted with caution, especially in relation to the associations between clinical risk phenotypes and the outcomes investigated. Finally, our analyses on OAC discontinuation and on the secondary outcomes should be regarded as exploratory, not being adjusted for the risk of competing events.

## CONCLUSIONS

In patients with AF, clinical risk phenotypes are heterogeneous, with more complex phenotypes associated with detrimental effect on thromboembolic prevention and risk of adverse outcomes. The magnitude of the effects increased with the increasing burden of complexity factors. Further efforts may be required to identify prevention strategies to improve prognosis in patients with AF with complex clinical phenotypes.

## Sources of Funding

This study was funded by Boehringer Ingelheim GmbH. The authors are solely responsible for the design and conduct of this study, all study analyses, the drafting and editing of the manuscript, and its final contents.

## Disclosures

Dr Romiti reports consultancy for Boehringer Ingelheim and an educational grant from Anthos. No fees are directly received personally. Dr Proietti is a national leader of the AFFIRMO project on multimorbidity in atrial fibrillation, which has received funding from the European Union's Horizon 2020 research and innovation program under grant agreement 899871. Dr Boriani received small speaker's fees from Medtronic, Boston, Boehringer Ingelheim, and Bayer, outside the submitted work. Dr Huisman has been receiving research grants from the Dutch Healthcare Fund, Dutch Heart Foundation, BMS‐Pfizer, Bayer Healthcare, and Boehringer Ingelheim; and consulting fees from BMS‐Pfizer, Bayer Healthcare, and Boehringer Ingelheim. Dr Lip has been consultant and speaker for BMS/Pfizer, Boehringer Ingelheim, Anthos, and Daiichi‐Sankyo. No fees are directly received personally. Dr Lip is a National Institute for Health and Care Research senior investigator and coprincipal investigator of the AFFIRMO project on multimorbidity in atrial fibrillation, which has received funding from the European Union's Horizon 2020 research and innovation program under grant agreement 899871. The remaining authors have no disclosures to report.

## Supporting information

Data S1
Tables S1–S3
Figures S1–S16
Reference ^44^
