# Supplementary material for: Implications of Clinical Risk Phenotypes on the Management and Natural History of Atrial Fibrillation: A Report From the GLORIA‐AF
Source: J Am Heart Assoc. 2023 Oct 17;12(20):e030565. doi: 10.1161/JAHA.123.030565 (PMC10757542; doi:10.1161/JAHA.123.030565)
Supplement: Supplementary file 1 — Data S1 Tables S1–S3 Figures S1–S16 Reference 44 [file JAH3-12-e030565-s001.pdf]

Supplemental Material

Data S1. GLORIA-AF Investigators

Dzifa Wosornu Abban
Nasser Abdul
Atilio Marcelo Abud
Fran Adams
Srinivas Addala
Pedro Adragão
Walter Ageno
Rajesh Aggarwal
Sergio Agosti
Piergiuseppe Agostoni
Francisco Aguilar
Julio Aguilar Linares
Luis Aguinaga
Jameel Ahmed
Allessandro Aiello
Paul Ainsworth
Jorge Roberto Aiub
Raed Al-Dallow
Lisa Alderson
Jorge Antonio Aldrete Velasco
Dimitrios Alexopoulos
Fernando Alfonso Manterola
Pareed Aliyar
David Alonso
Fernando Augusto Alves da Costa
José Amado
Walid Amara
Mathieu Amelot
Nima Amjadi
Fabrizio Ammirati
Marianna Andrade
Nabil Andrawis
Giorgio Annoni
Gerardo Ansalone
M.Kevin Ariani
Juan Carlos Arias
Sébastien Armero
Chander Arora
Muhammad Shakil Aslam
M. Asselman
Philippe Audouin
Charles Augenbraun
S. Aydin
Ivaneta Ayryanova
Emad Aziz
Luciano Marcelo Backes
E. Badings
Ermentina Bagni
Seth H. Baker
Richard Bala
Antonio Baldi
Shigenobu Bando
Subhash Banerjee
Alan Bank
Gonzalo Barón Esquivias
Craig Barr
Maria Bartlett
Vanja Basic Kes
Giovanni Baula
Steffen Behrens
Alan Bell
Raffaella Benedetti
Juan Benezet Mazuecos
Bouziane Benhalima
Jutta Bergler-Klein
Jean-Baptiste Berneau
Richard A. Bernstein
Percy Berrospi
Sergio Berti
Andrea Berz
Elizabeth Best
Paulo Bettencourt
Robert Betzu
Ravi Bhagwat
Luna Bhatta
Francesco Biscione
Giovanni Bisignani
Toby Black
Michael J. Bloch
Stephen Bloom
Edwin Blumberg
Mario Bo
Ellen Bøhmer
Andreas Bollmann
Maria Grazia Bongiorni
Giuseppe Boriani
D.J. Boswijk
Jochen Bott
Edo Bottacchi
Marica Bracic Kalan
Drew Bradman
Donald Brautigam
Nicolas Breton
P.J.A.M. Brouwers
Kevin Browne
Jordi Bruguera Cortada
A. Bruni
Claude Brunschwig
Hervé Buathier
Aurélie Buhl
John Bullinga
Jose Walter Cabrera
Alberto Caccavo
Shanglang Cai
Sarah Caine
Leonardo Calò
Valeria Calvi
Mauricio Camarillo Sánchez
Rui Candeias
Vincenzo Capuano
Alessandro Capucci
Ronald Caputo
Tatiana Cárdenas Rizo
Francisco Cardona
Francisco Carlos da Costa Darrieux
Yan Carlos Duarte Vera
Antonio Carolei
Susana Carreño
Paula Carvalho
Susanna Cary
Gavino Casu
Claudio Cavallini
Guillaume Cayla
Aldo Celentano
Tae-Joon Cha
Kwang Soo Cha
Jei Keon Chae
Kathrine Chalamidas
Krishnan Challappa
Sunil Prakash Chand
Harinath Chandrashekar
Ludovic Chartier
Kausik Chatterjee
Carlos Antero Chavez Ayala
Aamir Cheema
Amjad Cheema
Lin Chen

Table S1 – Baseline Characteristics of the Included Patients according to the Groups of Clinical Risk Phenotypes

Variable, n (%)	Groups of Clinical Risk Phenotypes								
	CHA_2DS_2-VASc ≥2* (n=14021)	CHA_2DS_2-VASc <2 (n=4073)	CKD Group (n=942)	Elderly Group (n=3563)	Stroke Group (n=2558)	Bleeding Group (n=746)	Stroke and Bleeding Group (n=433)	Multiple Features Group (n=2555)	p
Age, mean (SD)	68.5 (7.9)	57.9 (9.0)	70.9 (7.0)	83.3 (2.6)	68.7 (8.0)	70.0 (7.4)	73.9 (9.1)	82.2 (5.4)	<0.001
Female Sex	7035/14021 (50.2)	652/4073 (16.0)	478/942 (50.7)	2041/3563 (57.3)	1010/2558 (39.5)	342/746 (45.8)	187/433 (43.2)	1411/2555 (55.2)	<0.001
BMI, mean (SD)	29.3 (6.5)	28.5 (6.5)	30.5 (8.0)	26.5 (5.0)	28.2 (5.9)	29.6 (6.8)	27.5 (5.3)	27.0 (5.5)	<0.001
**Region**									<0.001
North America	3262/14021 (23.3)	924/4073 (22.7)	301/942 (32.0)	821/3563 (23.0)	423/2558 (16.5)	208/746 (27.9)	105/433 (24.2)	599/2555 (23.4)	
Europe	6812/14021 (48.6)	1742/4073 (42.8)	401/942 (42.6)	1975/3563 (55.4)	1386/2558 (54.2)	379/746 (50.8)	225/433 (52.0)	1510/2555 (59.1)	
Latin America	743/14021 (5.3)	172/4073 (4.2)	76/942 (8.1)	214/3563 (6.0)	110/2558 (4.3)	46/746 (6.2)	15/433 (3.5)	142/2555 (5.6)	
Africa/Middle East	250/14021 (1.8)	49/4073 (1.2)	32/942 (3.4)	41/3563 (1.2)	57/2558 (2.2)	5/746 (0.7)	5/433 (1.2)	38/2555 (1.5)	
Asia	2954/14021 (21.1)	1186/4073 (29.1)	132/942 (14.0)	512/3563 (14.4)	582/2558 (22.8)	108/746 (14.5)	83/433 (19.2)	266/2555 (10.4)	
**AF Type**									<0.001
Paroxysmal AF	7768/14021 (55.4)	2406/4073 (59.1)	522/942 (55.4)	1754/3563 (49.2)	1551/2558 (60.6)	428/746 (57.4)	241/433 (55.7)	1297/2555 (50.8)	
Persistent AF	5059/14021 (36.1)	1460/4073 (35.8)	328/942 (34.8)	1289/3563 (36.2)	750/2558 (29.3)	256/746 (34.3)	134/433 (30.9)	851/2555 (33.3)	
Permanent AF	1194/14021 (8.5)	207/4073 (5.1)	92/942 (9.8)	520/3563 (14.6)	257/2558 (10.0)	62/746 (8.3)	58/433 (13.4)	407/2555 (15.9)	
**Symptoms**									<0.001
EHRA I	3937/12140 (32.4)	1055/3565 (29.6)	304/816 (37.3)	1206/3114 (38.7)	1206/2322 (51.9)	219/630 (34.8)	206/388 (53.1)	1134/2251 (50.4)	
EHRA II	5187/12140 (42.7)	1681/3565 (47.2)	304/816 (37.3)	1274/3114 (40.9)	762/2322 (32.8)	251/630 (39.8)	117/388 (30.2)	727/2251 (32.3)	
EHRA III	2381/12140 (19.6)	658/3565 (18.5)	172/816 (21.1)	478/3114 (15.4)	265/2322 (11.4)	125/630 (19.8)	38/388 (9.8)	294/2251 (13.1)	
EHRA IV	635/12140 (5.2)	171/3565 (4.8)	36/816 (4.4)	156/3114 (5.0)	89/2322 (3.8)	35/630 (5.6)	27/388 (7.0)	96/2251 (4.3)	
**Medical History**									
Arterial Hypertension	11171/14000 (79.8)	1982/4060 (48.8)	816/941 (86.7)	2698/3562 (75.7)	1890/2555 (74.0)	606/743 (81.6)	344/432 (79.6)	2112/2551 (82.8)	<0.001
CHF	3618/13939 (26.0)	477/4050 (11.8)	378/932 (40.6)	938/3535 (26.5)	423/2536 (16.7)	188/735 (25.6)	81/427 (19.0)	703/2524 (27.9)	<0.001
CAD	2890/13723 (21.1)	331/4002 (8.3)	280/917 (30.5)	746/3475 (21.5)	519/2509 (20.7)	208/720 (28.9)	112/417 (26.9)	659/2496 (26.4)	<0.001
Diabetes Mellitus	3968/14021 (28.3)	143/4073 (3.5)	418/942 (44.4)	686/3563 (19.3)	601/2558 (23.5)	206/746 (27.6)	121/433 (27.9)	679/2555 (26.6)	<0.001
PAD	412/13917 (3.0)	15/4071 (0.4)	51/935 (5.5)	119/3528 (3.4)	91/2539 (3.6)	41/736 (5.6)	27/428 (6.3)	147/2522 (5.8)	<0.001
History of Stroke/TIA	0/14021 (0.0)	0/4073 (0.0)	0/942 (0.0)	0/3563 (0.0)	2558/2558 (100.0)	0/746 (0.0)	433/433 (100.0)	1421/2555 (55.6)	<0.001

	Groups of Clinical Risk Phenotypes								
**Variable, n (%)**	**CHA_2DS_2-VASc ≥2* (n=14021)**	**CHA_2DS_2-VASc <2 (n=4073)**	**CKD Group (n=942)**	**Elderly Group (n=3563)**	**Stroke Group (n=2558)**	**Bleeding Group (n=746)**	**Stroke and Bleeding Group (n=433)**	**Multiple Features Group (n=2555)**	**p**
Previous Bleeding	0/14021 (0.0)	139/4073 (3.4)	0/942 (0.0)	0/3563 (0.0)	0/2558 (0.0)	746/746 (100.0)	433/433 (100.0)	404/2555 (15.8)	<0.001
CKD	0/14021 (0.0)	63/4073 (1.5)	942/942 (100.0)	0/3563 (0.0)	0/2558 (0.0)	0/746 (0.0)	44/433 (10.2)	1281/2555 (50.1)	<0.001
COPD	921/13964 (6.6)	131/4062 (3.2)	93/934 (10.0)	278/3556 (7.8)	157/2555 (6.1)	62/742 (8.4)	31/430 (7.2)	212/2544 (8.3)	<0.001
Dementia	34/13970 (0.2)	1/4066 (0.0)	3/941 (0.3)	48/3551 (1.4)	13/2540 (0.5)	1/741 (0.1)	10/429 (2.3)	74/2547 (2.9)	<0.001
Neoplasia	1278/13932 (9.2)	212/4045 (5.2)	106/938 (11.3)	482/3542 (13.6)	229/2544 (9.0)	118/739 (16.0)	66/431 (15.3)	367/2542 (14.4)	<0.001
**Scores**									
CHA2DS2-VASc, mean (SD)	3.0 ± 1.0	1.0 ± 0.0	3.7 ± 1.2	3.9 ± 1.0	4.7 ± 1.3	3.1 ± 1.1	5.3 ± 1.3	5.2 ± 1.4	<0.001
HAS-BLED, mean (SD)	1.2 ± 0.8	0.7 ± 0.7	1.6 ± 0.8	1.4 ± 0.6	2.0 ± 0.9	2.3 ± 0.8	3.2 ± 0.8	2.2 ± 0.9	<0.001
**Antithrombotic Treatment**									<0.001
Antiplatelets	1404/14019 (10.0)	732/4072 (18.0)	109/942 (11.6)	357/3563 (10.0)	278/2555 (10.9)	74/746 (9.9)	69/433 (15.9)	302/2554 (11.8)	
NOAC	8109/14019 (57.8)	1939/4072 (47.6)	404/942 (42.9)	1998/3563 (56.1)	1516/2555 (59.3)	405/746 (54.3)	214/433 (49.4)	1275/2554 (49.9)	
VKA	3722/14019 (26.5)	886/4072 (21.8)	372/942 (39.5)	1003/3563 (28.2)	627/2555 (24.5)	218/746 (29.2)	96/433 (22.2)	803/2554 (31.4)	
None	784/14019 (5.6)	515/4072 (12.6)	57/942 (6.1)	205/3563 (5.8)	134/2555 (5.2)	49/746 (6.6)	54/433 (12.5)	174/2554 (6.8)	
**Other Treatments**									
ACE	4764/14021 (34.0)	908/4073 (22.3)	306/942 (32.5)	1008/3563 (28.3)	812/2558 (31.7)	261/746 (35.0)	142/433 (32.8)	832/2555 (32.6)	<0.001
ARB	3741/14021 (26.7)	629/4073 (15.4)	254/942 (27.0)	949/3563 (26.6)	577/2558 (22.6)	214/746 (28.7)	96/433 (22.2)	603/2555 (23.6)	<0.001
Statins	6217/14021 (44.3)	1065/4073 (26.1)	500/942 (53.1)	1420/3563 (39.9)	1705/2558 (66.7)	373/746 (50.0)	296/433 (68.4)	1441/2555 (56.4)	<0.001
Beta-Blockers	9267/14021 (66.1)	2395/4073 (58.8)	664/942 (70.5)	2101/3563 (59.0)	1522/2558 (59.5)	522/746 (70.0)	256/433 (59.1)	1566/2555 (61.3)	<0.001

Legend: *Patients with CHA_2DS_2-VASc ≥2 and without any other feature. ACEi= Angiotensin Converting Enzyme inhibitors; ARB= Angiotensin-II Receptor Blockers; BMI= Body Mass Index; CAD= Coronary Artery Disease; CHF= Congestive Heart Failure; CKD= Chronic Kidney Disease COPD= Chronic Obstructive Pulmonary Disease; EHRA= European Heart Rhythm Association; IQR= Interquartile Range; NOAC= Non-Vitamin K antagonist Oral Anticoagulant; PAD= Peripheral Artery Disease; SD= Standard Deviation; TIA= Transient Ischemic Attack VKA= Vitamin K Antagonist.

Table S2 – Baseline Characteristics according to patients included/excluded from the analysis on the primary composite outcome

Variable, n (%)	Included (n=20521)	Excluded (n=8370)	p
Age, mean (SD)	70.0 (10.3)	70.1 (11.0)	0.478
Female Sex	9262/20521 (45.1)	3894/8370 (46.5)	0.032
BMI, mean (SD)	28.7 (6.4)	28.4 (6.3)	0.001
Enrolled in Phase III	16772/20521 (81.7)	76/8370 (0.9)	<0.001
**Region**			<0.001
North America	4675/20521 (22.8)	1968/8370 (23.5)	
Europe	10521/20521 (51.3)	3909/8370 (46.7)	
Latin America	1199/20521 (5.8)	319/8370 (3.8)	
Africa/Middle East	267/20521 (1.3)	210/8370 (2.5)	
Asia	3859/20521 (18.8)	1964/8370 (23.5)	
**AF Type**			0.019
Paroxysmal AF	11447/20521 (55.8)	4520/8370 (54.0)	
Persistent AF	7099/20521 (34.6)	3028/8370 (36.2)	
Permanent AF	1975/20521 (9.6)	822/8370 (9.8)	
**Symptoms**			<0.001
EHRA I	6621/19383 (34.2)	2646/5843 (45.3)	
EHRA II	7142/19383 (36.8)	3161/5843 (54.1)	
EHRA III	4385/19383 (22.6)	26/5843 (0.4)	
EHRA IV	1235/19383 (6.4)	10/5843 (0.2)	
**Medical History**			
Arterial Hypertension	15453/20496 (75.4)	6166/8348 (73.9)	0.006
CHF	4684/20381 (23.0)	2122/8297 (25.6)	<0.001
CAD	3938/20084 (19.6)	1807/8175 (22.1)	<0.001
Diabetes Mellitus	4821/20521 (23.5)	2001/8370 (23.9)	0.452
PAD	601/20381 (2.9)	302/8295 (3.6)	0.002
History of Stroke/TIA	3157/20521 (15.4)	1255/8370 (15.0)	0.403
Previous Bleeding	1183/20521 (5.8)	539/8370 (6.4)	0.028
CKD	1501/20521 (7.3)	829/8370 (9.9)	<0.001
COPD	1294/20454 (6.3)	591/8333 (7.1)	0.017
Dementia	125/20459 (0.6)	59/8326 (0.7)	0.346
Neoplasia	2034/20393 (10.0)	824/8320 (9.9)	0.857
**Scores**			
CHA2DS2-VASc, mean (SD)	3.2 ± 1.5	3.3 ± 1.6	0.005
HAS-BLED, mean (SD)	1.4 ± 0.9	1.5 ± 0.9	<0.001
**Antithrombotic Treatment**			<0.001
Antiplatelets	1843/20514 (9.0)	1482/8370 (17.7)	
NOAC	13848/20514 (67.5)	2012/8370 (24.0)	
VKA	3773/20514 (18.4)	3954/8370 (47.2)	
None	1050/20514 (5.1)	922/8370 (11.0)	
**Other Treatments**			
ACE	6378/20521 (31.1)	2655/8370 (31.7)	0.287
ARB	5166/20521 (25.2)	1897/8370 (22.7)	<0.001
Statins	9406/20521 (45.8)	3611/8370 (43.1)	<0.001
Beta-Blockers	13171/20521 (64.2)	5122/8370 (61.2)	<0.001

Legend: ACEi= Angiotensin Converting Enzyme inhibitors; ARB= Angiotensin-II Receptor Blockers; BMI= Body Mass Index; CAD= Coronary Artery Disease; CHF= Congestive Heart Failure; CKD= Chronic Kidney Disease COPD= Chronic Obstructive Pulmonary Disease; EHRA= European Heart

Rhythm Association; IQR= Interquartile Range; NOAC= Non-Vitamin K antagonist Oral Anticoagulant; PAD= Peripheral Artery Disease; SD= Standard Deviation; TIA= Transient Ischemic Attack VKA= Vitamin K Antagonist.

Table S3 – Baseline Characteristics according to the Burden of Clinical Risk Features

Variable, n (%)	No Feature (n=18094)	1 Feature (n=7809)	2 Feature (n=2542)	3+ Features (n=446)	p
Age, mean (SD)	66.1 ± 9.3	75.5 ± 9.2	80.6 ± 7.1	83.3 ± 4.2	<0.001
Female Sex	7687/18094 (42.5)	3871/7809 (49.6)	1343/2542 (52.8)	255/446 (57.2)	<0.001
BMI, mean (SD)	29.2 (6.5)	27.8 (6.1)	27.2 (5.5)	26.8 (5.1)	<0.001
**Region**					<0.001
North America	4186/18094 (23.1)	1753/7809 (22.4)	590/2542 (23.2)	114/446 (25.6)	
Europe	8554/18094 (47.3)	4141/7809 (53.0)	1479/2542 (58.2)	256/446 (57.4)	
Latin America	915/18094 (5.1)	446/7809 (5.7)	139/2542 (5.5)	18/446 (4.0)	
Africa/Middle East	299/18094 (1.7)	135/7809 (1.7)	39/2542 (1.5)	4/446 (0.9)	
Asia	4140/18094 (22.9)	1334/7809 (17.1)	295/2542 (11.6)	54/446 (12.1)	
**AF Type**					<0.001
Paroxysmal AF	10174/18094 (56.2)	4255/7809 (54.5)	1305/2542 (51.3)	233/446 (52.2)	
Persistent AF	6519/18094 (36.0)	2623/7809 (33.6)	849/2542 (33.4)	136/446 (30.5)	
Permanent AF	1401/18094 (7.7)	931/7809 (11.9)	388/2542 (15.3)	77/446 (17.3)	
**Symptoms**					<0.001
EHRA I	4992/15705 (31.8)	2935/6882 (42.6)	1132/2245 (50.4)	208/394 (52.8)	
EHRA II	6868/15705 (43.7)	2591/6882 (37.6)	719/2245 (32.0)	125/394 (31.7)	
EHRA III	3039/15705 (19.4)	1040/6882 (15.1)	287/2245 (12.8)	45/394 (11.4)	
EHRA IV	806/15705 (5.1)	316/6882 (4.6)	107/2245 (4.8)	16/394 (4.1)	
**Medical History**					
Arterial Hypertension	13153/18060 (72.8)	6010/7801 (77.0)	2076/2537 (81.8)	380/446 (85.2)	<0.001
CHF	4095/17989 (22.8)	1927/7738 (24.9)	670/2510 (26.7)	114/441 (25.9)	<0.001
CAD	3221/17725 (18.2)	1753/7621 (23.0)	647/2478 (26.1)	124/435 (28.5)	<0.001
Diabetes Mellitus	4111/18094 (22.7)	1911/7809 (24.5)	661/2542 (26.0)	139/446 (31.2)	<0.001
PAD	427/17988 (2.4)	302/7738 (3.9)	142/2509 (5.7)	32/441 (7.3)	<0.001
History of Stroke/TIA	0/18094 (0.0)	2558/7809 (32.8)	1476/2542 (58.1)	378/446 (84.8)	<0.001
Previous Bleeding	139/18094 (0.8)	746/7809 (9.6)	603/2542 (23.7)	234/446 (52.5)	<0.001
CKD	63/18094 (0.3)	942/7809 (12.1)	1001/2542 (39.4)	324/446 (72.6)	<0.001
COPD	1052/18026 (5.8)	590/7787 (7.6)	213/2530 (8.4)	30/444 (6.8)	<0.001

Variable, n (%)	No Feature (n=18094)	1 Feature (n=7809)	2 Feature (n=2542)	3+ Features (n=446)	p
Dementia	35/18036 (0.2)	65/7773 (0.8)	65/2532 (2.6)	19/444 (4.3)	<0.001
Neoplasia	1490/17977 (8.3)	935/7763 (12.0)	356/2528 (14.1)	77/445 (17.3)	<0.001
**Scores**					
CHA2DS2-VASc, mean (SD)	2.6 ± 1.2	4.1 ± 1.2	5.0 ± 1.3	5.9 ± 1.2	<0.001
HAS-BLED, mean (SD)	1.1 ± 0.8	1.7 ± 0.8	2.2 ± 0.9	2.9 ± 0.9	<0.001
**Antithrombotic Treatment**					<0.001
Antiplatelets	2136/18091 (11.8)	818/7806 (10.5)	309/2541 (12.2)	62/446 (13.9)	
NOAC	10048/18091 (55.5)	4323/7806 (55.4)	1293/2541 (50.9)	196/446 (43.9)	
VKA	4608/18091 (25.5)	2220/7806 (28.4)	754/2541 (29.7)	145/446 (32.5)	
None	1299/18091 (7.2)	445/7806 (5.7)	185/2541 (7.3)	43/446 (9.6)	
**Other Treatments**					
ACE	5672/18094 (31.3)	2387/7809 (30.6)	839/2542 (33.0)	135/446 (30.3)	0.132
ARB	4370/18094 (24.2)	1994/7809 (25.5)	588/2542 (23.1)	111/446 (24.9)	0.040
Statins	7282/18094 (40.2)	3998/7809 (51.2)	1467/2542 (57.7)	270/446 (60.5)	<0.001
Beta-Blockers	11662/18094 (64.5)	4809/7809 (61.6)	1550/2542 (61.0)	272/446 (61.0)	<0.001

Legend: ACEi= Angiotensin Converting Enzyme inhibitors; ARB= Angiotensin-II Receptor Blockers; BMI= Body Mass Index; CAD= Coronary Artery Disease; CHF= Congestive Heart Failure; CKD= Chronic Kidney Disease COPD= Chronic Obstructive Pulmonary Disease; EHRA= European Heart Rhythm Association; IQR= Interquartile Range; NOAC= Non-Vitamin K antagonist Oral Anticoagulant; PAD= Peripheral Artery Disease; SD= Standard Deviation; TIA= Transient Ischemic Attack VKA= Vitamin K Antagonist.

Figure S1. Relationship between Features, Groups, and Burden of Clinical Risk Phenotypes.

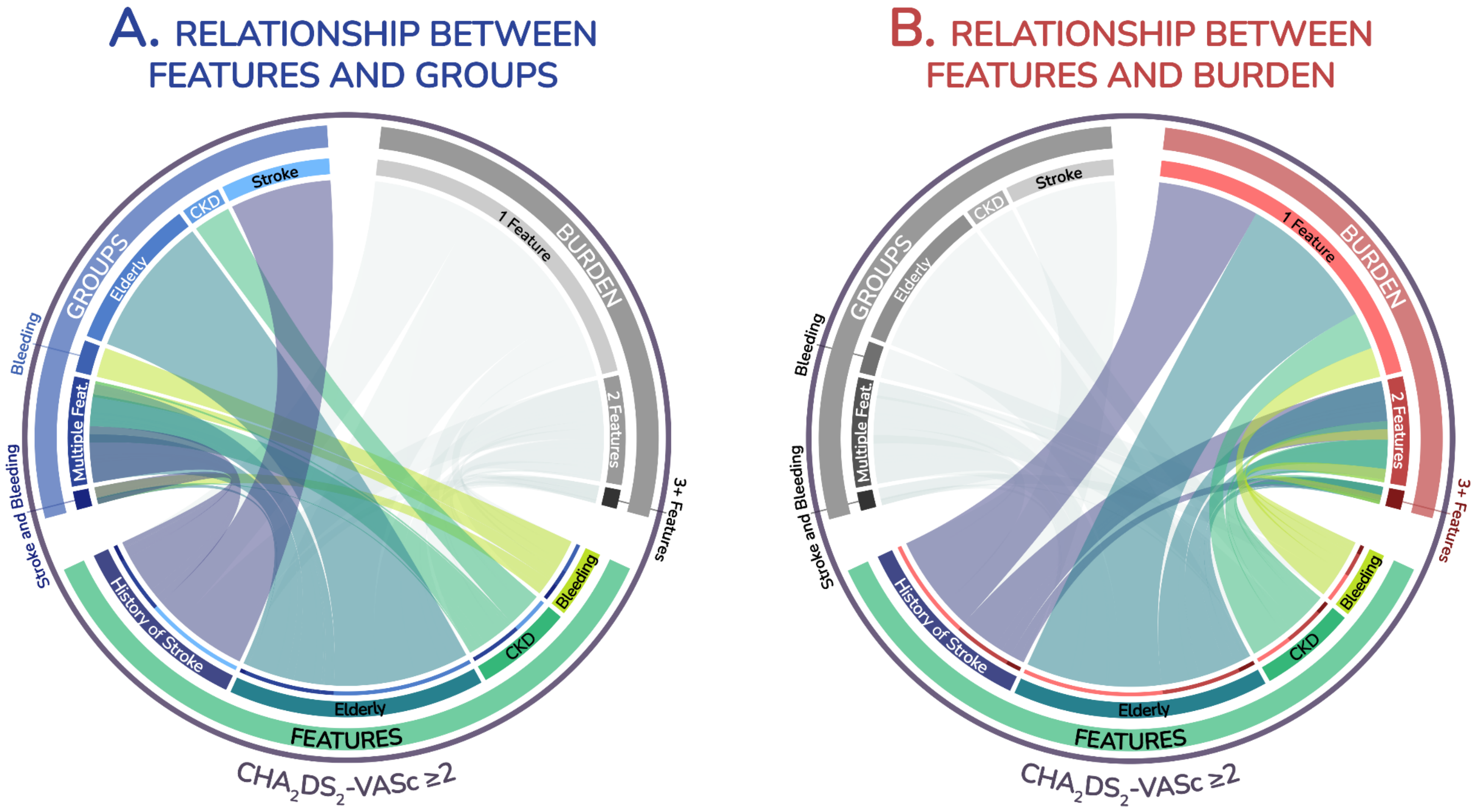

Legend: Panel A: Relationship between Features and Groups; Panel B: Relationship between Features and Burden. CKD= Chronic Kidney Disease

Figure S2 – Contribution of each Feature to Groups and Burden

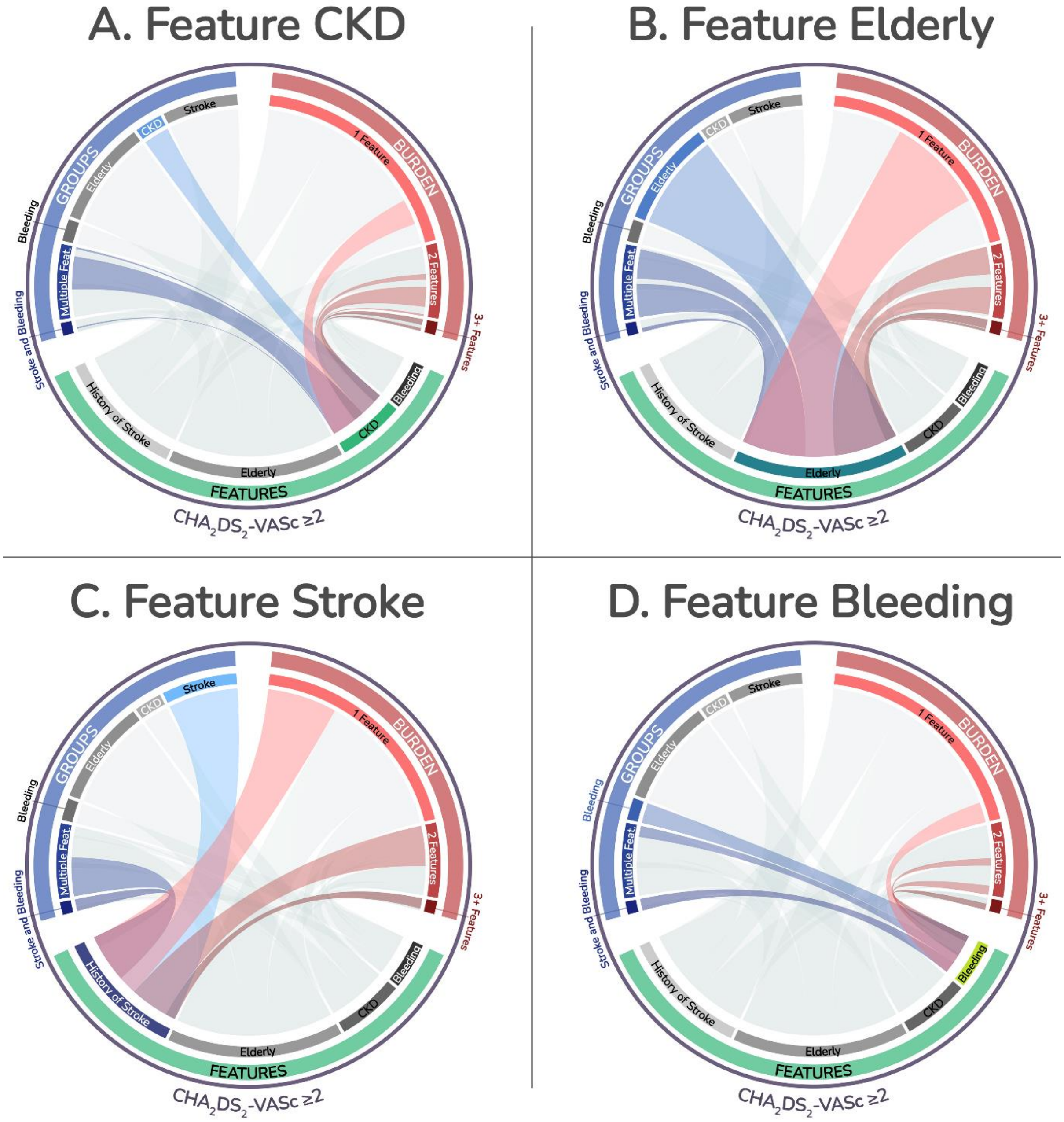

Legend: Panel A: Feature CKD; Panel B: Feature Elderly; Panel C: Feature Stroke; Panel D: Feature Bleeding. CKD= Chronic Kidney Disease

Figure S3 – Combinations of Features into Groups and Burden

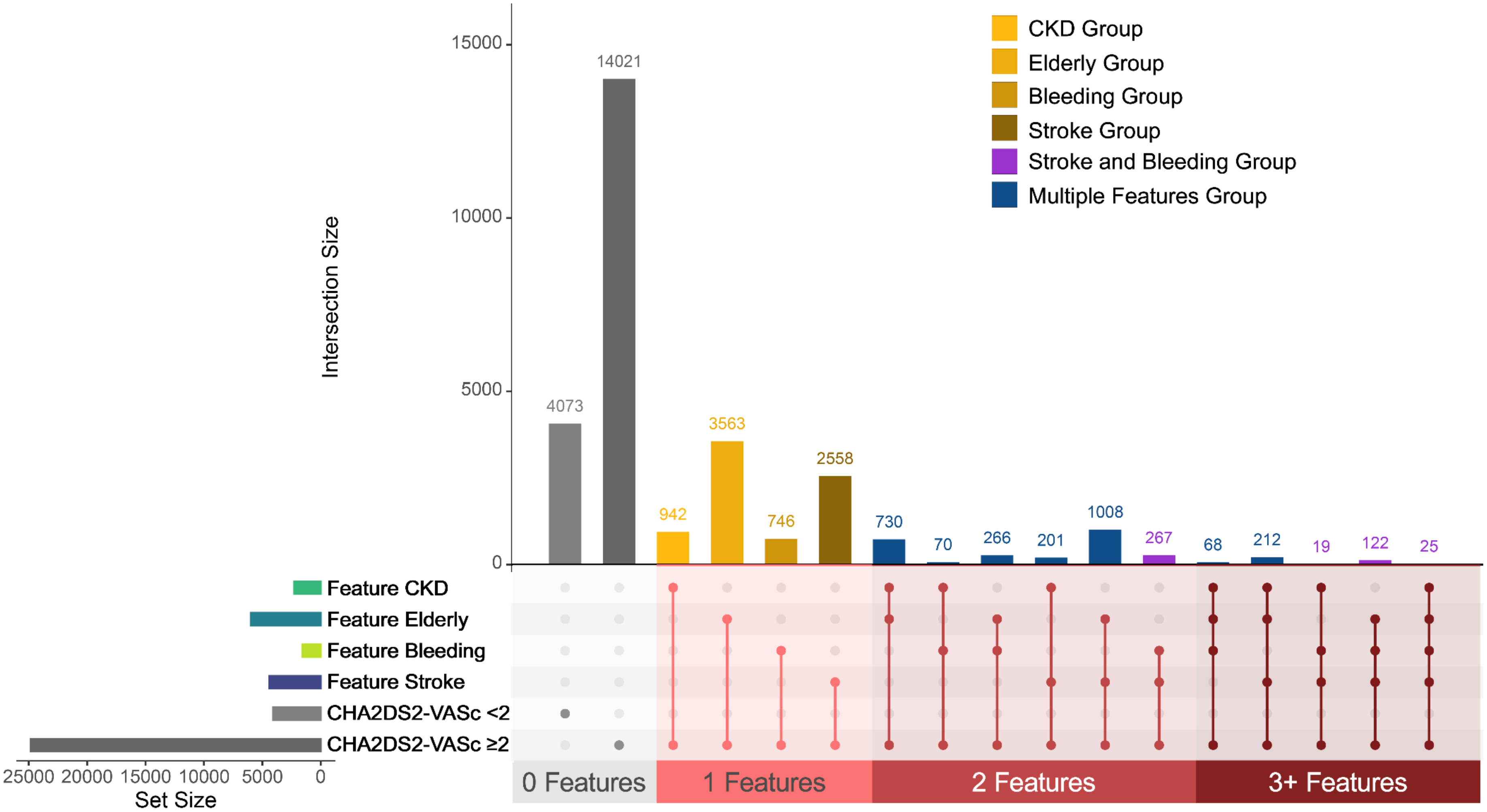

Legend: Horizontal bar colors represent different features; Vertical bar colors represent different groups; Shades of red represent different burden. CKD= Chronic Kidney Disease. Figure created using the UpSetR[44] package in R

Figure S4 – Antithrombotic Treatment at Baseline according to groups of clinical risk phenotypes

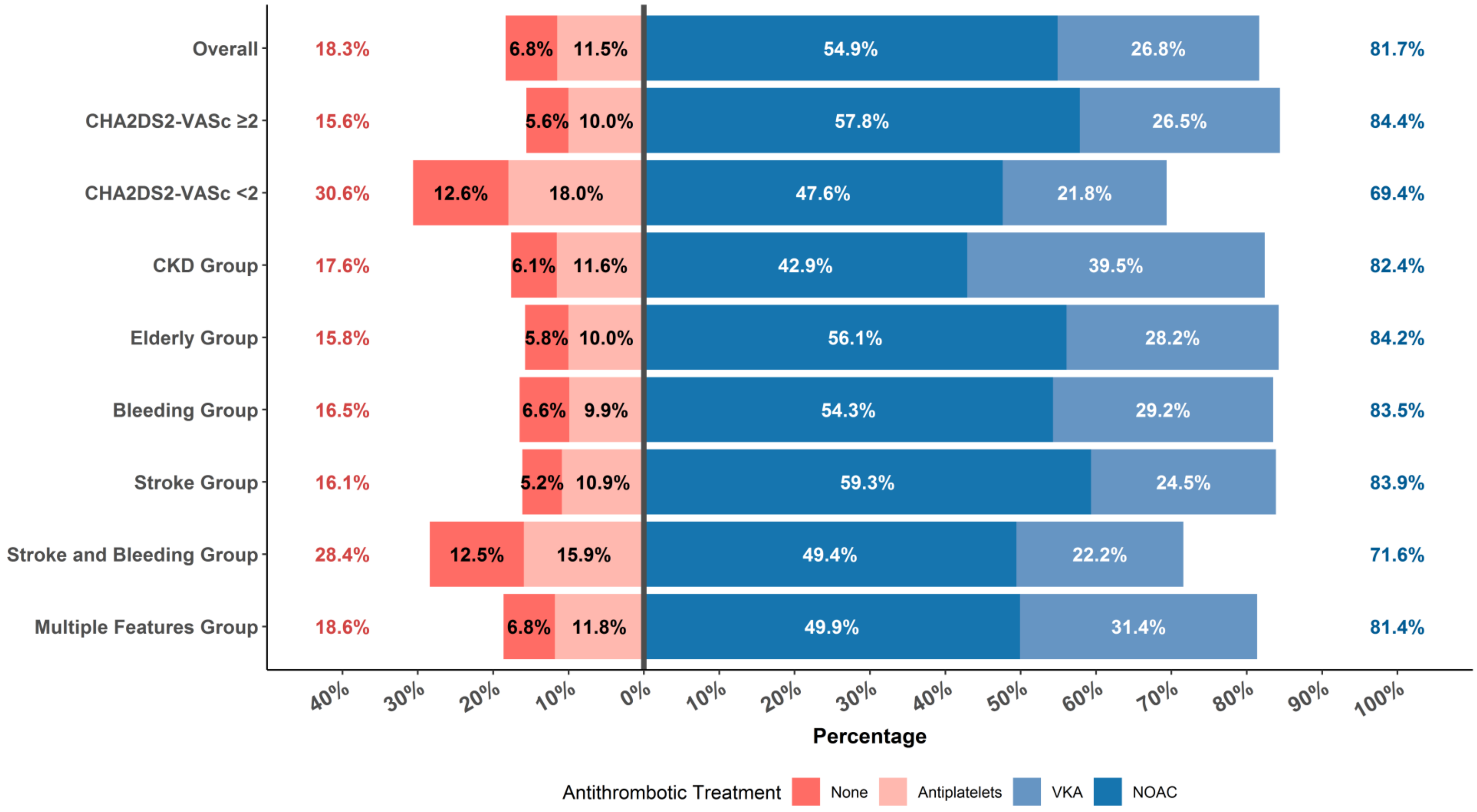

Legend: CKD= Chronic Kidney Disease; NOAC= Non-vitamin K oral anticoagulant; VKA= Vitamin K antagonist

Figure S5 – Rates of OAC Persistence and Discontinuation at 6, 12 and 24 months, according to groups of clinical risk phenotypes

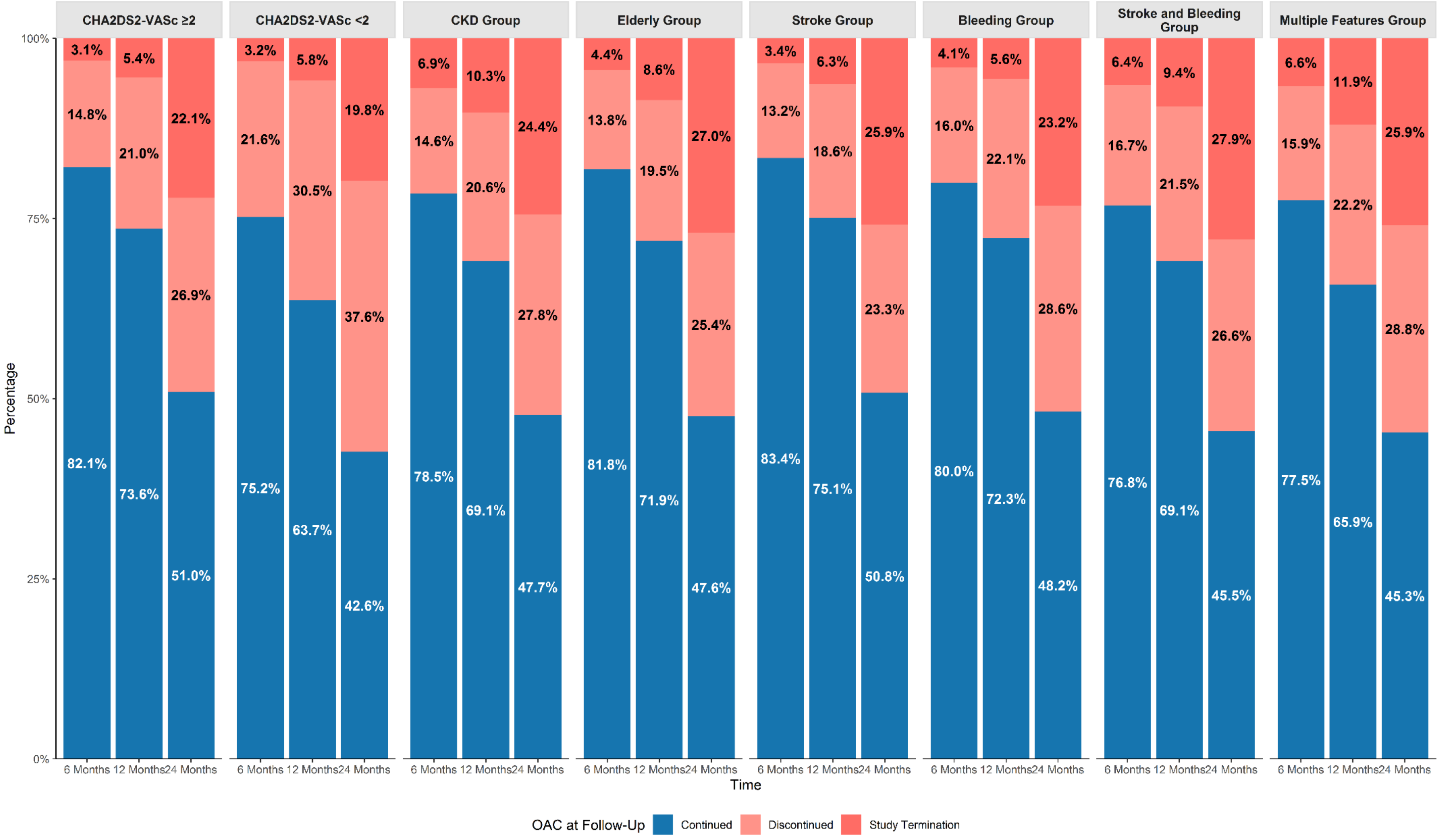

Legend: CKD= Chronic Kidney Disease; OAC= Oral Anticoagulant

Figure S6 – Rates of NOAC (Panel A) and VKA (Panel B) Persistence and Discontinuation at 6, 12 and 24 months according to groups of clinical risk phenotypes

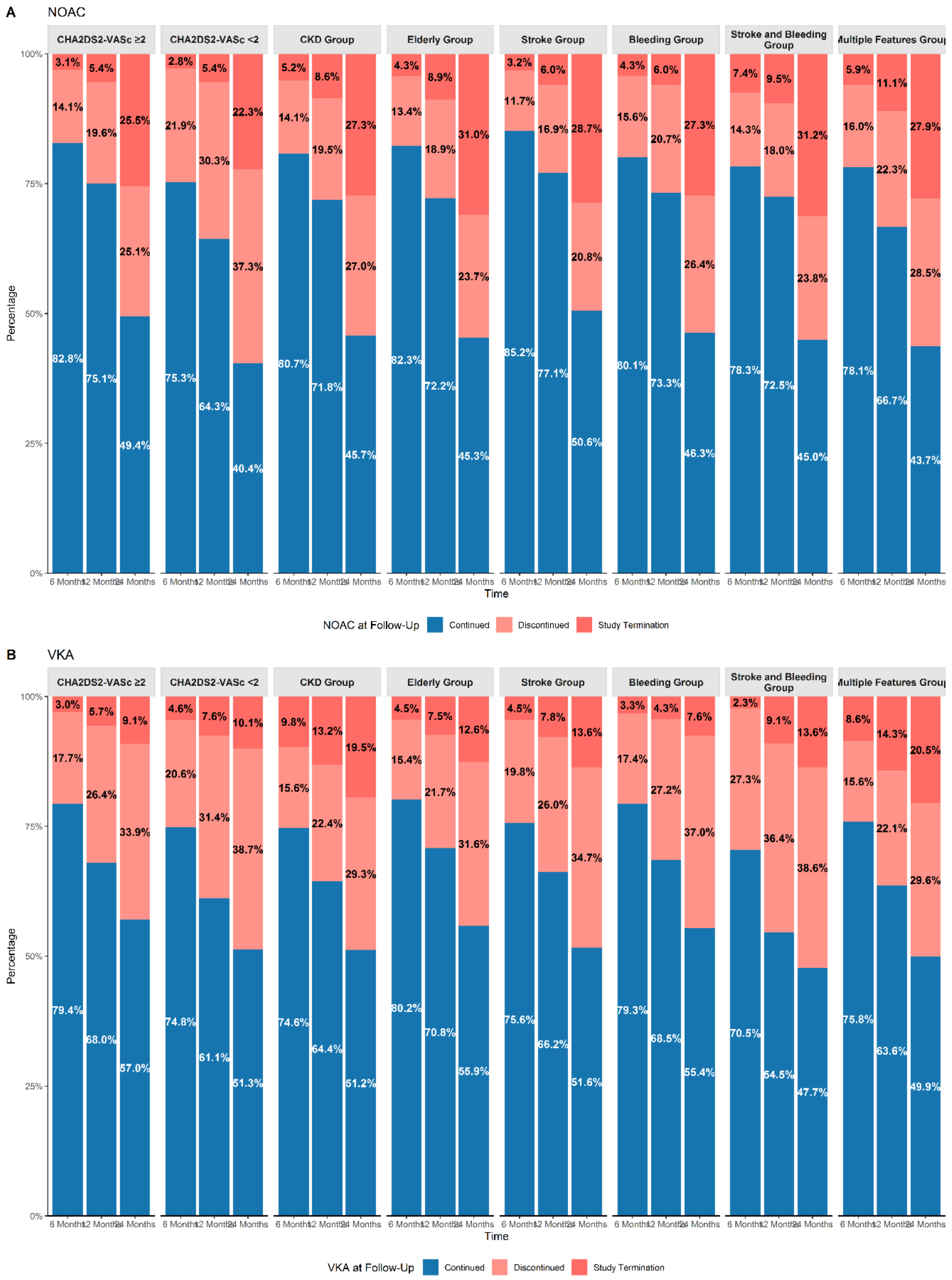

Legend: CKD= Chronic Kidney Disease; OAC= Oral Anticoagulant; NOAC= Non-Vitamin K Antagonist Oral Anticoagulant; VKA= Vitamin K Antagonist

Figure S7 – Cox-Regression on OAC discontinuation, stratified by NOAC (Panel A) and VKA (Panel B) use at baseline, according to groups of clinical risk phenotypes

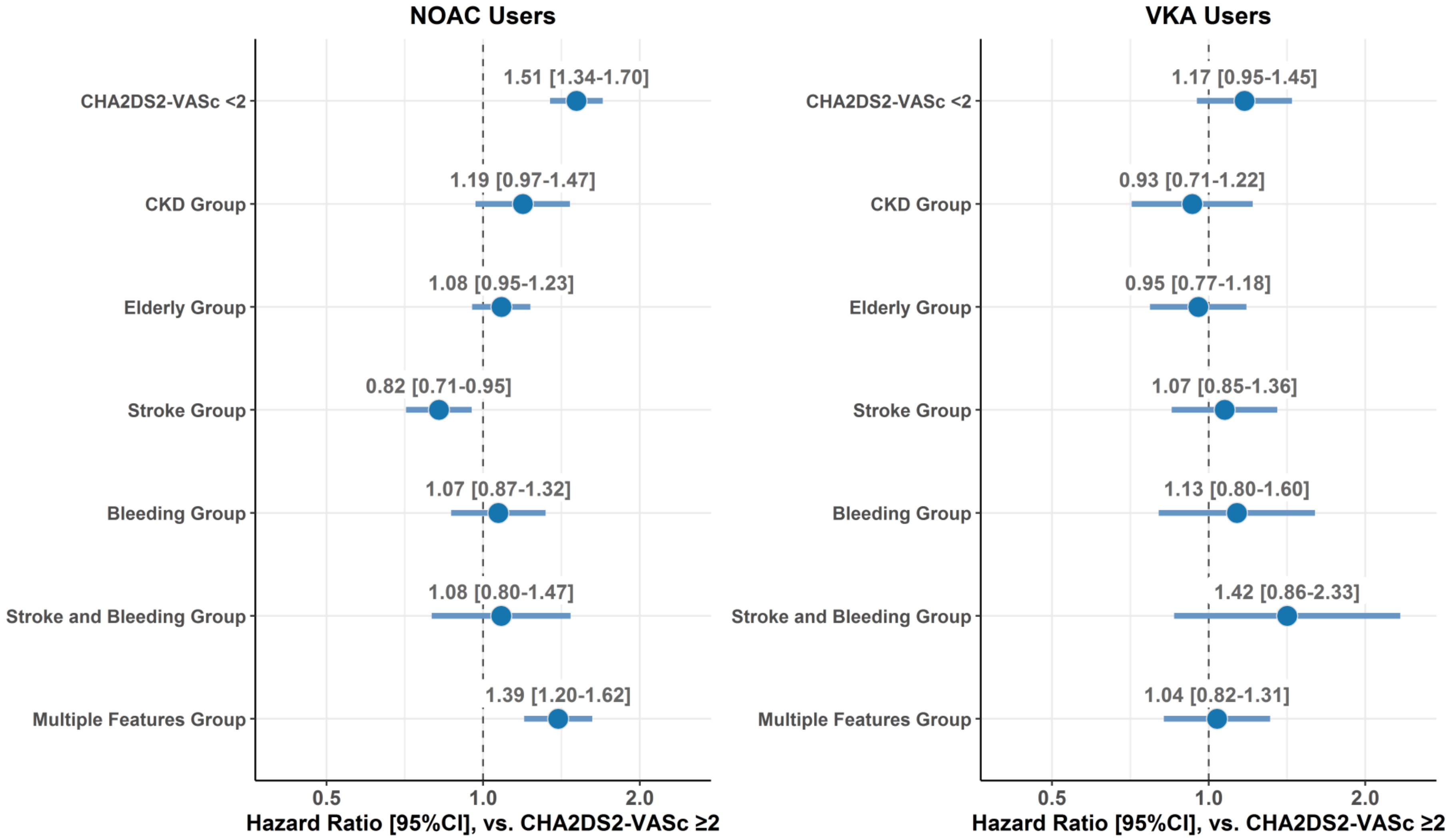

Legend: CKD= Chronic Kidney Disease; NOAC= Non vitamin-K antagonist oral anticoagulant; VKA= vitamin K antagonist

Figure S8 – Kaplan-Meier curves for the risk of the Primary Composite Outcome, according to group of clinical risk phenotypes

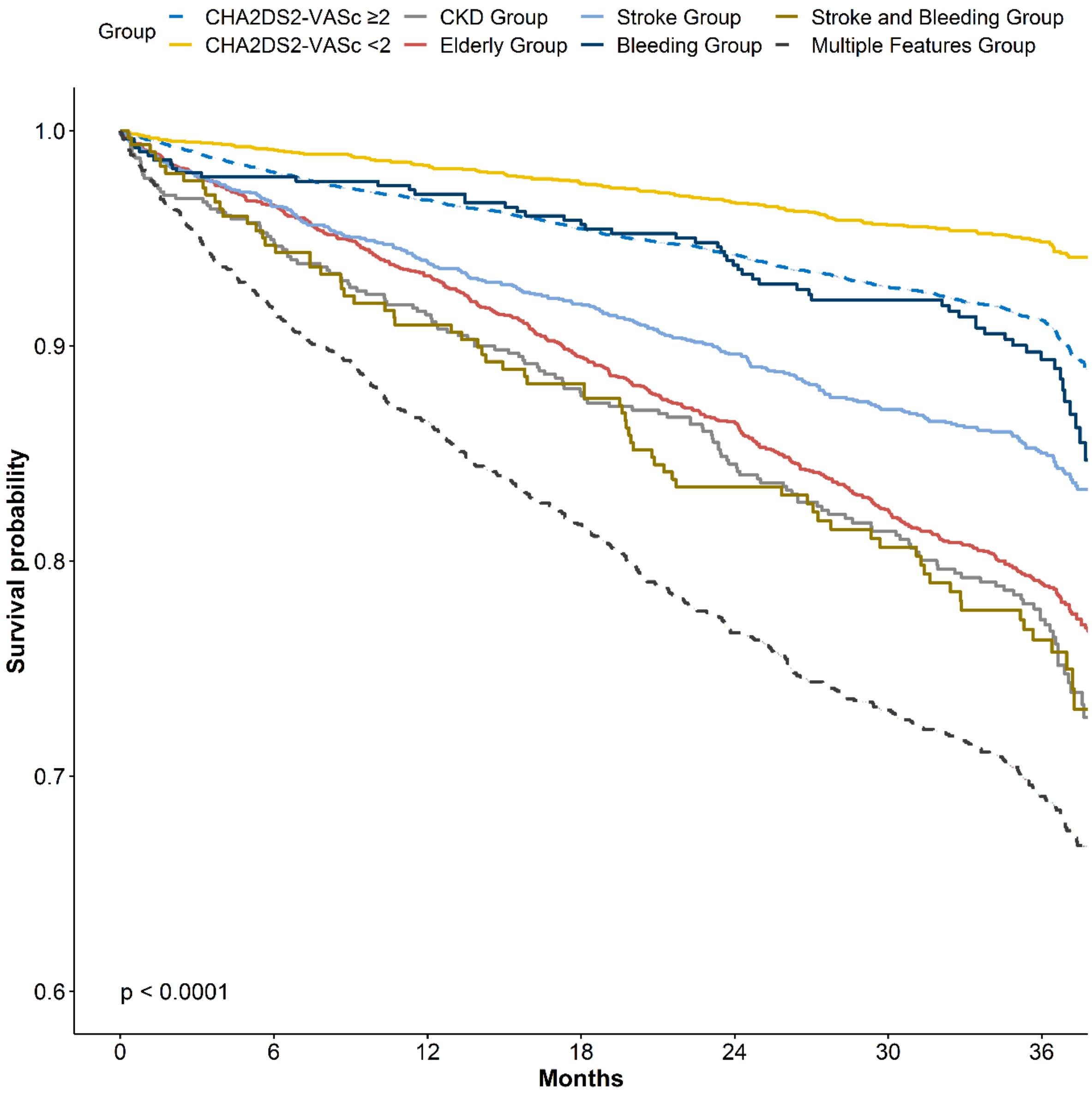

Number at risk

	0	6	12	18	24	30	36
	10168	9909	9648	9374	8900	7287	5680
	2846	2812	2753	2673	2570	2197	1717
	633	599	569	530	496	417	315
	2463	2354	2236	2102	1946	1587	1213
	1905	1827	1763	1705	1602	1262	1003
	512	494	485	468	436	362	261
	304	283	268	258	235	195	150
	1690	1540	1428	1323	1201	988	733

Months

Legend: CKD= Chronic Kidney Disease.

Figure S9 – Cumulative burden according to each feature

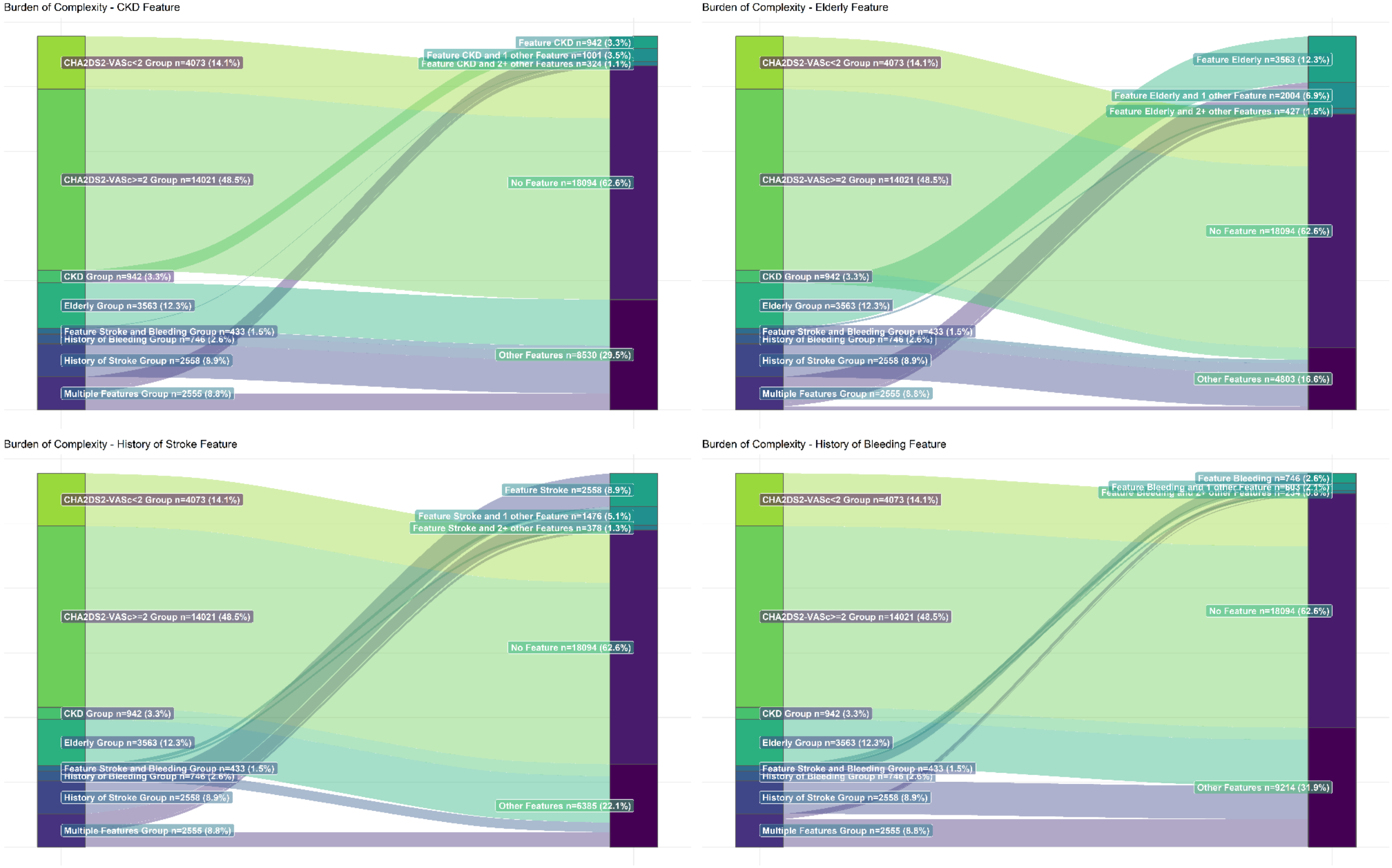

Legend: CKD= Chronic Kidney Disease

Figure S10 - Antithrombotic Treatment at Baseline according to the burden of features

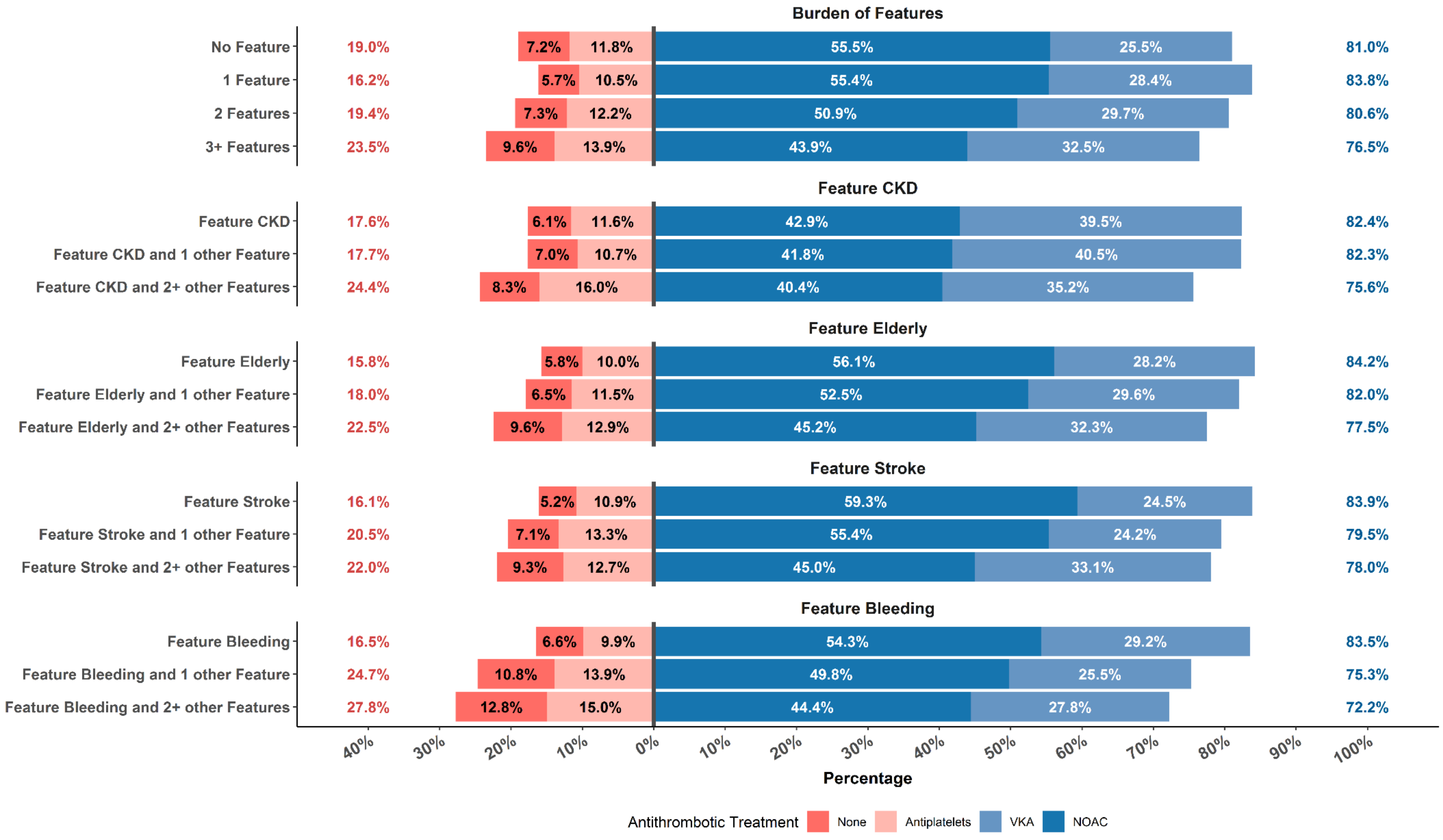

Legend: CKD= Chronic Kidney Disease; NOAC= Non-vitamin K oral anticoagulant; VKA= Vitamin K antagonist.

Figure S11 – Regression on use of OAC according to the burden of features

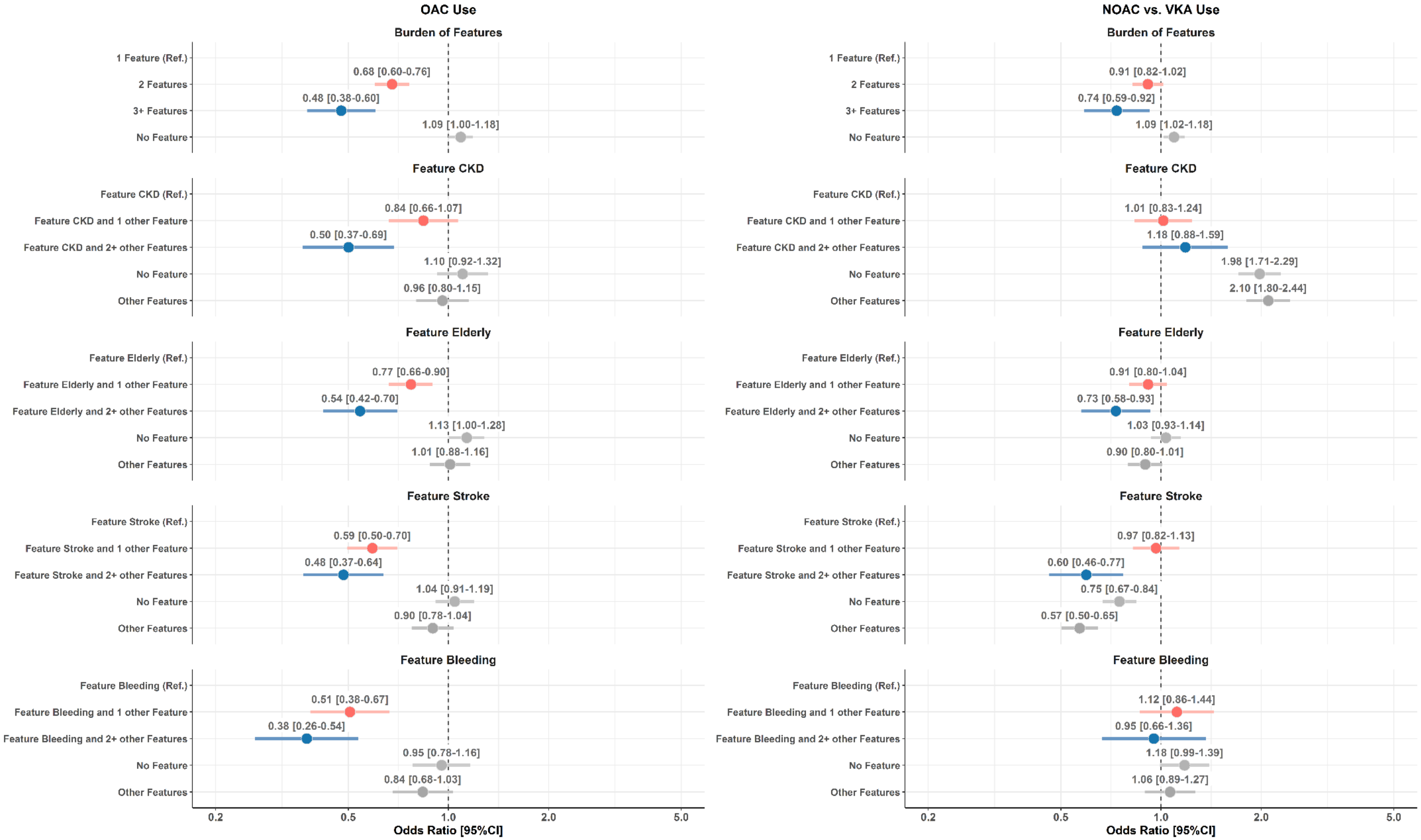

Legend: CKD= Chronic Kidney Disease; NOAC= Non-vitamin K oral anticoagulant; VKA= Vitamin K antagonist

Figure S12 – Rates of OAC Persistence and Discontinuation at 6, 12 and 24 months according to the burden of features

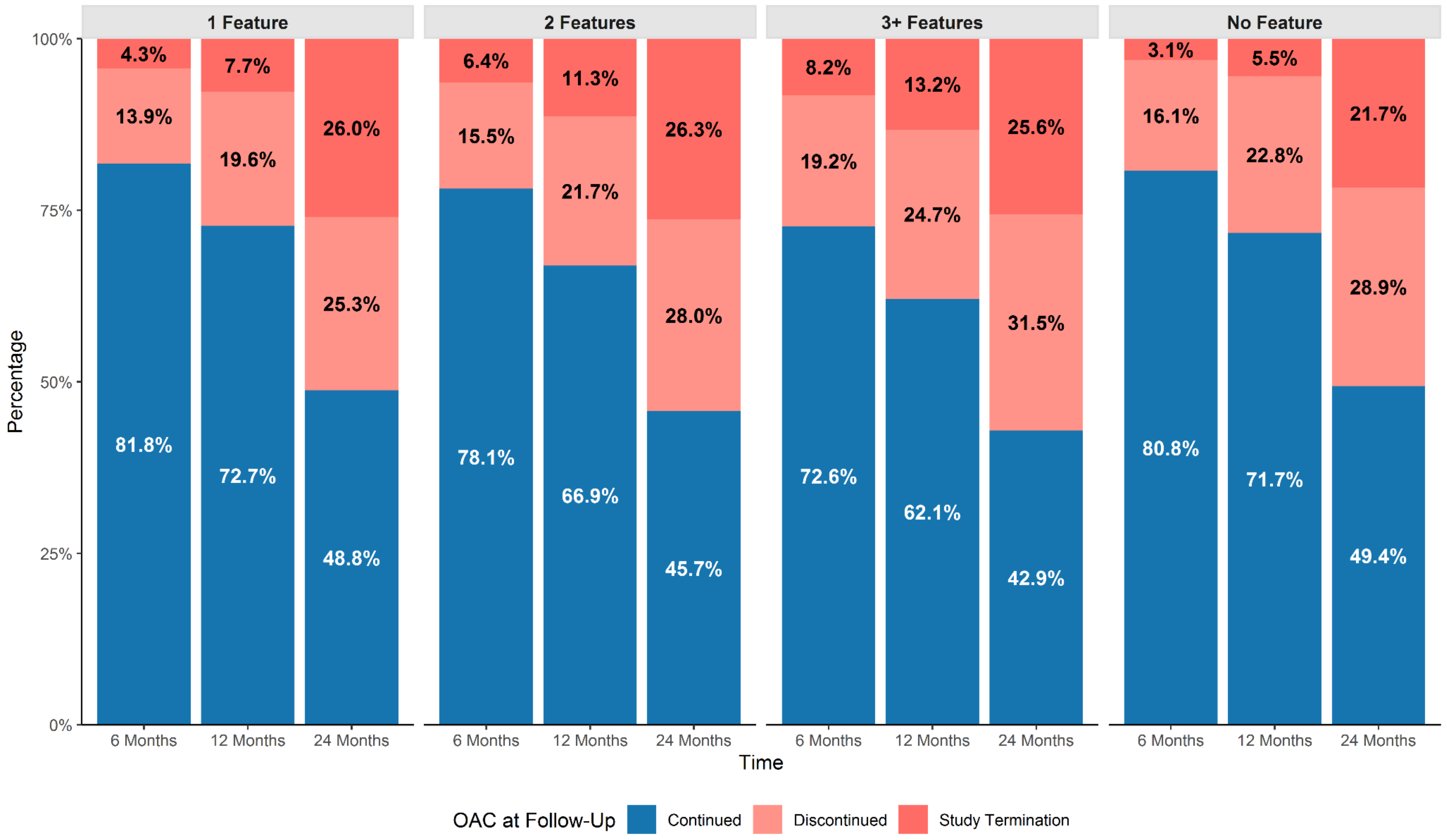

Legend: CKD= Chronic Kidney Disease

Figure S13 – Rates of OAC Persistence and Discontinuation at 6, 12 and 24 months according to the burden of features

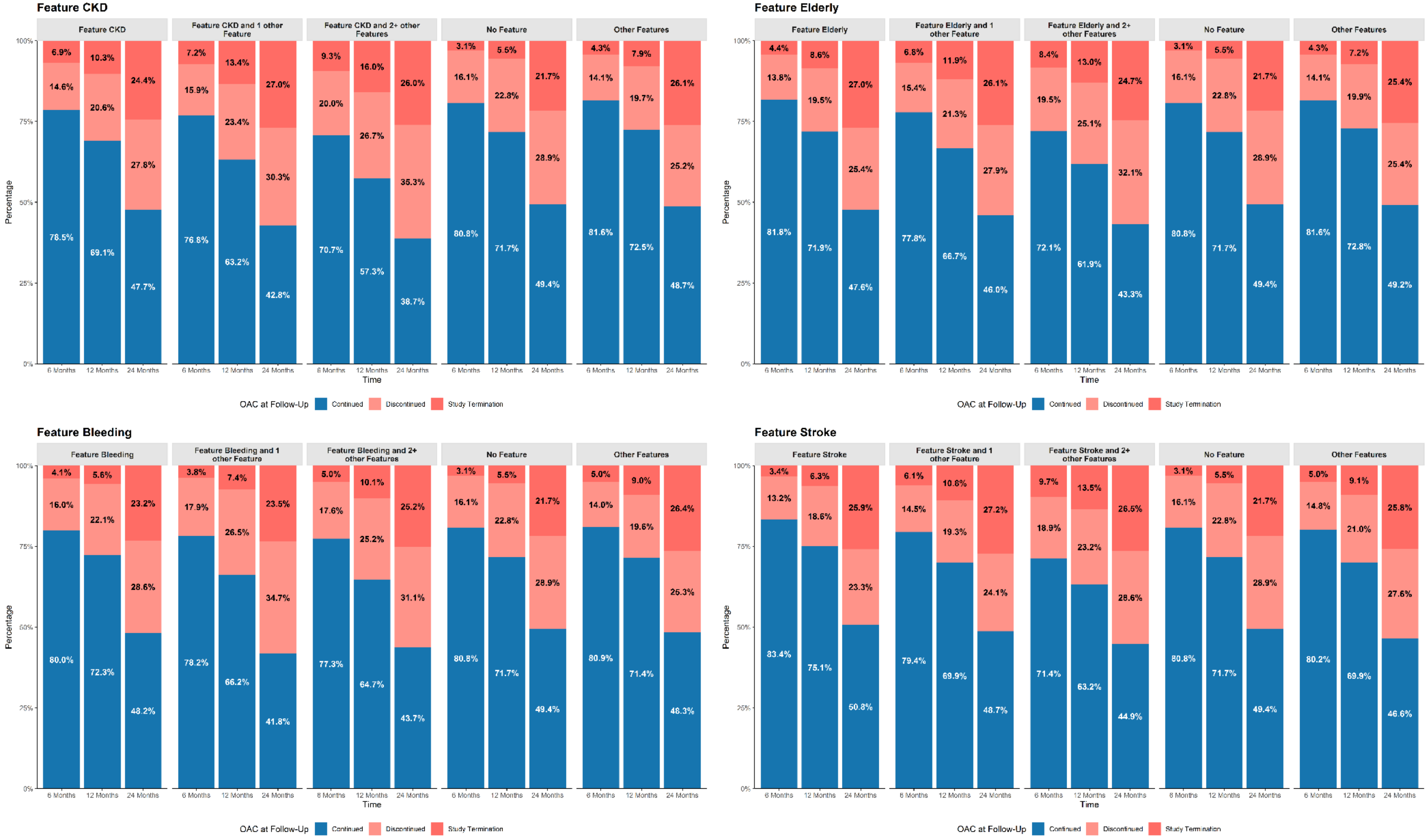

Legend: CKD= Chronic Kidney Disease

Figure S14 - Cox Regression on OAC Discontinuation according to the burden of features

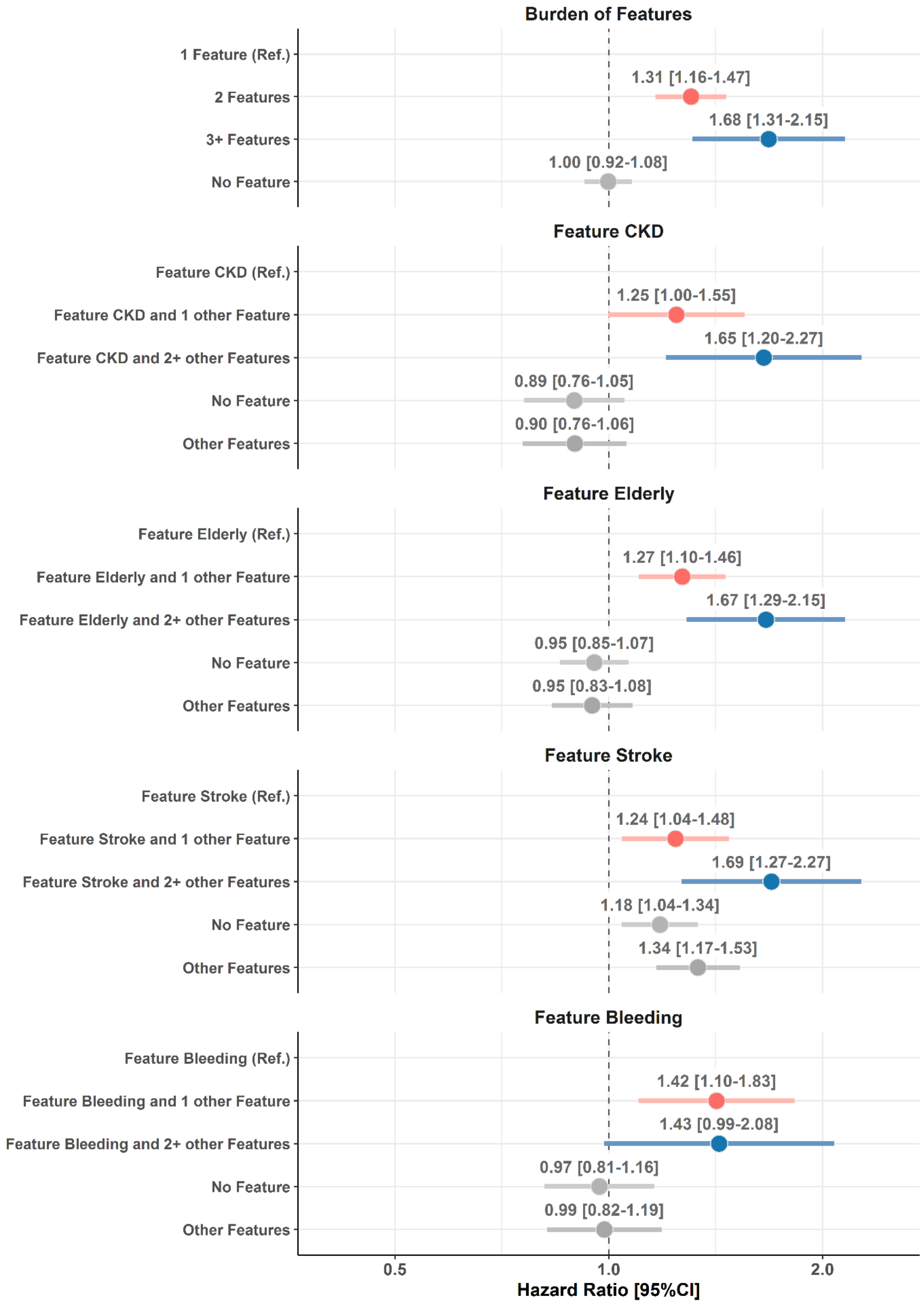

Legend: CKD= Chronic Kidney Disease

Figure S15 – Kaplan-Meier curves for the Primary Composite Outcome according to the burden of features

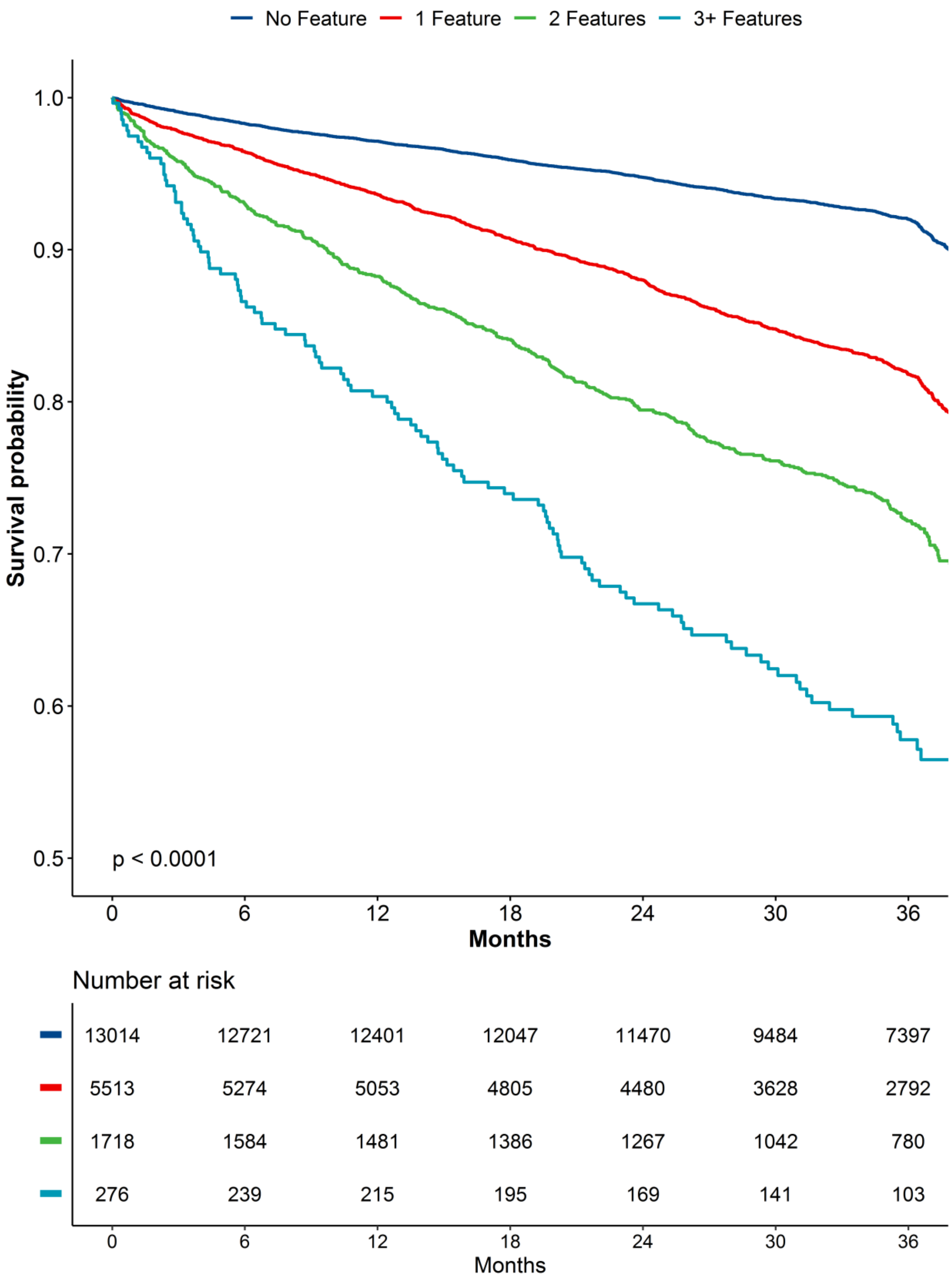

Figure S16 – Cox Regression Analysis for the Primary Composite Outcome according to the burden of features

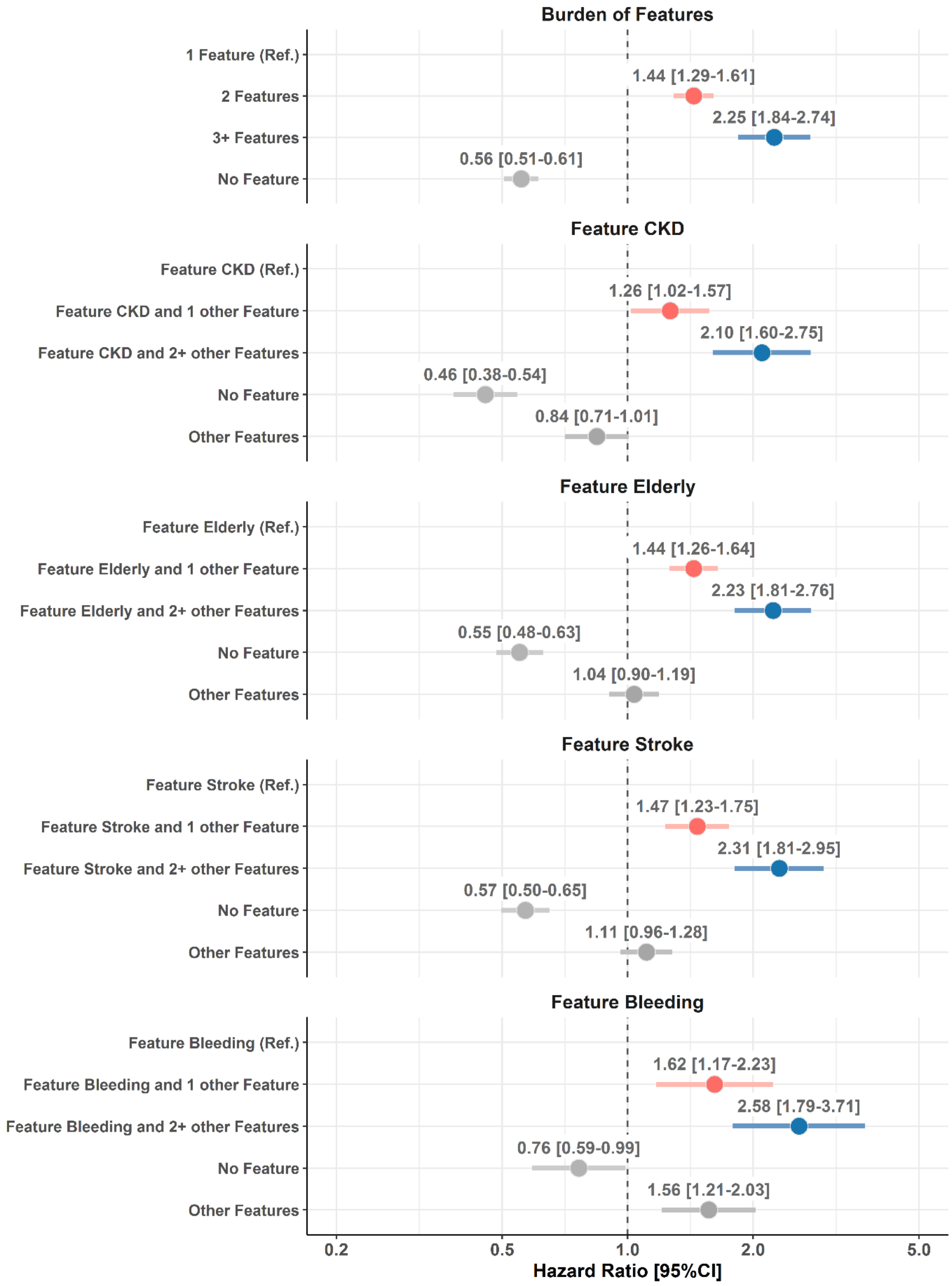

Legend: CKD= Chronic Kidney Disease
